# Exosome-Mediated Crosstalk Between Tumor and Tumor-Associated Macrophages

**DOI:** 10.3389/fmolb.2021.764222

**Published:** 2021-10-13

**Authors:** Qi Chen, Yuefeng Li, Wujiang Gao, Lu Chen, Wenlin Xu, Xiaolan Zhu

**Affiliations:** ^1^ Department of Oncology and Central Laboratory, Fourth Affiliated Hospital of Jiangsu University, Zhenjiang, China; ^2^ International Genome Center, Jiangsu University, Zhenjiang, China; ^3^ Affiliated People Hospital of Jiangsu University, Zhenjiang, China; ^4^ Reproduction Medicine Center, Fourth Affiliated Hospital of Jiangsu University, Zhenjiang, China

**Keywords:** exosomes, tumor microenvironment, cargoes delivery, immunotherapy, tumor associated macrophages

## Abstract

Exosomes are nanosized vesicles, derived from the endolysosomal compartment of cells and can shuttle diverse biomolecules such as nucleic acids, proteins, lipids, amino acids, and metabolites, which can reflect their origin cells. Delivery of these cargoes to recipient cells enables exosomes to influence diverse cellular functions. As one of the most abundant immune cells in the tumor microenvironment, tumor-associated macrophages (TAMs) are educated by the tumor milieu, which is rich in cancer cells and stroma components, to exert functions such as the promotion of tumor growth, immunosuppression, angiogenesis, and cancer cell dissemination. Herein, we focus on exosomes-mediated intercellular communication between tumor cells and TAM in the tumor microenvironment, which may provide new targets for anti-tumor treatment. In this review, we highlight the most recent studies on the effect of tumor/macrophage-derived exosomes on macrophage/tumor function in different cancer types.

## Introduction

Although the study of extacellular vesicles (EVs) is continuously evolving, they generally divide into two major categories, exosomes (40–160 nm) and ectosomes (50 nm-1 μm in diameter, including microvesicles, microparticles and large vesicles) (Meldolesi, 2018; Kalluri and LeBleu, 2020). Unlike ectosomes that fall off the surface of the plasma membrane via outward budding, exosomes are membrane-derived vesicles with a size range of −40–160 nm (average −100 nm) in diameter with an endosome origin that can be released by all types of cells. Depending on the cell of origin, exosomes contain many constituents of a cell, including DNA, RNA, lipids, metabolites, and cytosolic and surface proteins (Kalluri and LeBleu, 2020). It has been demonstrated that exosomes are associated with immune response and cancer progression (Li Z. et al., 2020; Ma et al., 2021; Schwarzenbach and Gahan, 2021). The components delivered by exosomes to recipient cells result in the alteration of biological response, which can be disease-promoting or restraining. These specific cellular components in exosomes have been indicated that they have a crucial role in regulating intercellular communication (Colombo et al., 2014; Devhare and Ray, 2018; Maia et al., 2018). The efficient delivery of certain components through exosomes can provide novel strategies in designing exosome-based therapeutics.

The tumor microenvironment (TME) plays a crucial role in tumor progression and metastasis. Macrophages, as one of the most abundant immune cells in the TME, exhibit noticeable phenotypic switch responding to divergent environmental conditions. The presence of macrophages across malignancies are in line with the established role of macrophages in tumorigenesis *in vivo*, from angiogenesis initiation to universal metastasis (Cortés et al., 2017). In addition, their phenotypes are defined as two polarized types based on the *in vitro* polarization of M0 macrophages, which are classically activated M1 macrophages and alternately activated M2 macrophages (the alternately activated macrophages are further subdivided into M2a, b, c and d). The macrophage activation is induced by many cytokines, including classical activation by LPS and IFN-γ, alternative activation by IL-4 and oxidized lipids (Mox)-induced phenotype (Orecchioni et al., 2019). Other cytokines such as IL-10 and TGF-β are also associated with macrophage polarization. Thus, these two phenotypes can be identified by their specific markers, metabolic features, and gene expression profiles (Garzetti et al., 2014; Wu et al., 2020). M1 macrophages exert anti-tumor effects, while M2 macrophages have anti-inflammatory and tumor-promoting properties (Garzetti et al., 2014). Those macrophages infiltrating tumor tissues or populating in the TME are termed tumor-associated macrophages (TAMs), including both resident macrophages and circulating monocytes recruited to the TME. TAMs, activated by IL-10 or TGF-β, display mostly M2 phenotype features and discharge pro-tumorigenic factors, chemokines and cytokines (Wu et al., 2020). Pathways and molecules involved in polarizing TAM are diverse in different tumors, including: IL-4 and IL-13, eosinophils (Eos) and basophils (Bas); cytokines and metabolites from cancer cells; antibodies (Ab) from B cells and immune complex; stromal cell-derived factors (IL-1, LT) (Mantovani et al., 2017). The presence of TAM infiltration in TME is crucial in promoting tumor development and metastasis by stimulating angiogenesis, tumor growth, migration and invasion, as well as in EMT and tumor resistance (Zhang et al., 2018). High level of TAMs infiltration is associated with cancer progression and poor overall survival rate of cancer patients (Zhang et al., 2018; Lin et al., 2019; Zhao Y. et al., 2019). Recent studies are emerging to identify novel therapeutic interventions targeting the destructive tumor-infiltrating myeloid cells, and the possible breakthrough lies probably in the crosstalk between tumor cells and TAMs, in which macrophages are induced to M2-like polarization and in turn support tumor growth.

Cancer immunotherapy is generally divided into two types: active and passive immunotherapy. Active immunotherapy involves decreasing cancer cells by activating the immune system, while passive immunotherapy involves the passive acceptance of antibodies, cytokines, or transformed immune cells that can directly act on the tumor (Zhang Z. et al., 2021). In addition, based on their capabilities to transfer molecules from their origin cells to peripheral circulation, increasing studies are considering exosomes as tumor biomarkers (Lan B. et al., 2019). Importantly, both natural and modified EVs offer the possibility of delivering the therapeutic immunology components for the control and destruction of tumor cells (Schwarzenbach and Gahan, 2021). Exosomes are less toxic and immunogenic than the other nano-carriers. Due to the presence of CD47 on their surface, exosomes can effectively avoid phagocytosis by the circulating monocytes, thus facilitating the transfer of the cargos (Kamerkar et al., 2017). Therefore, it is a promising and inspiring idea to use exosomes as vehicles, and modify exosomes for clinical applications through artificially optimizing the integration of specific loadings such as tumor drugs and targeting siRNA (Naseri et al., 2020; Zhan et al., 2020).

Tumor cells and tumor-associated cells, acting as either host cells or recipient cells, can transport biomolecules and cell components through exosomes (Schwarzenbach and Gahan, 2021). These exosomes are considered as new participants in the mechanism of tumor growth, invasion, angiogenesis, inflammation response, immunologic remodeling, and therapeutic effects, strongly supporting the significance of exosome-mediated host-recipient communication. Herein, we summarize important findings on the part of exosomes play in the communication between tumors and macrophages in different cancer types, emphasizing the crucial modulating role of this process in TME of tumor initiation, progression, metastasis and response to chemotherapy.

## The Close Relationship Between Tumor-Associated Macrophages and Tumor Progression

As immunosuppressive factors, TAMs express an M2-like phenotype, and they are recognized as important participants in fostering pre-metastatic niches, tumor progression and chemoresistance, as well as in providing soluble mediators for cells proliferation, metastasis, survival, and genetic instability (Mantovani et al., 2017; Petty and Yang, 2017; Lin et al., 2019), and in directly and indirectly suppressing the activity of cytotoxic T cells (Ruffell et al., 2012). TAM-derived neuropilin-2 (NRP2) stimulates tumor growth by regulating efferocytosis of apoptotic tumor cells and coordinating immune suppression (Roy et al., 2018). In addition, TAM-derived IL-10 promotes cancer stem cell (CSC)-like characteristics of non-small cell lung cancer (NSCLC) cells through JAK1/STAT1/NF-κB/Notch1 signaling (Yang L. et al., 2019).

**FIGURE 1:**
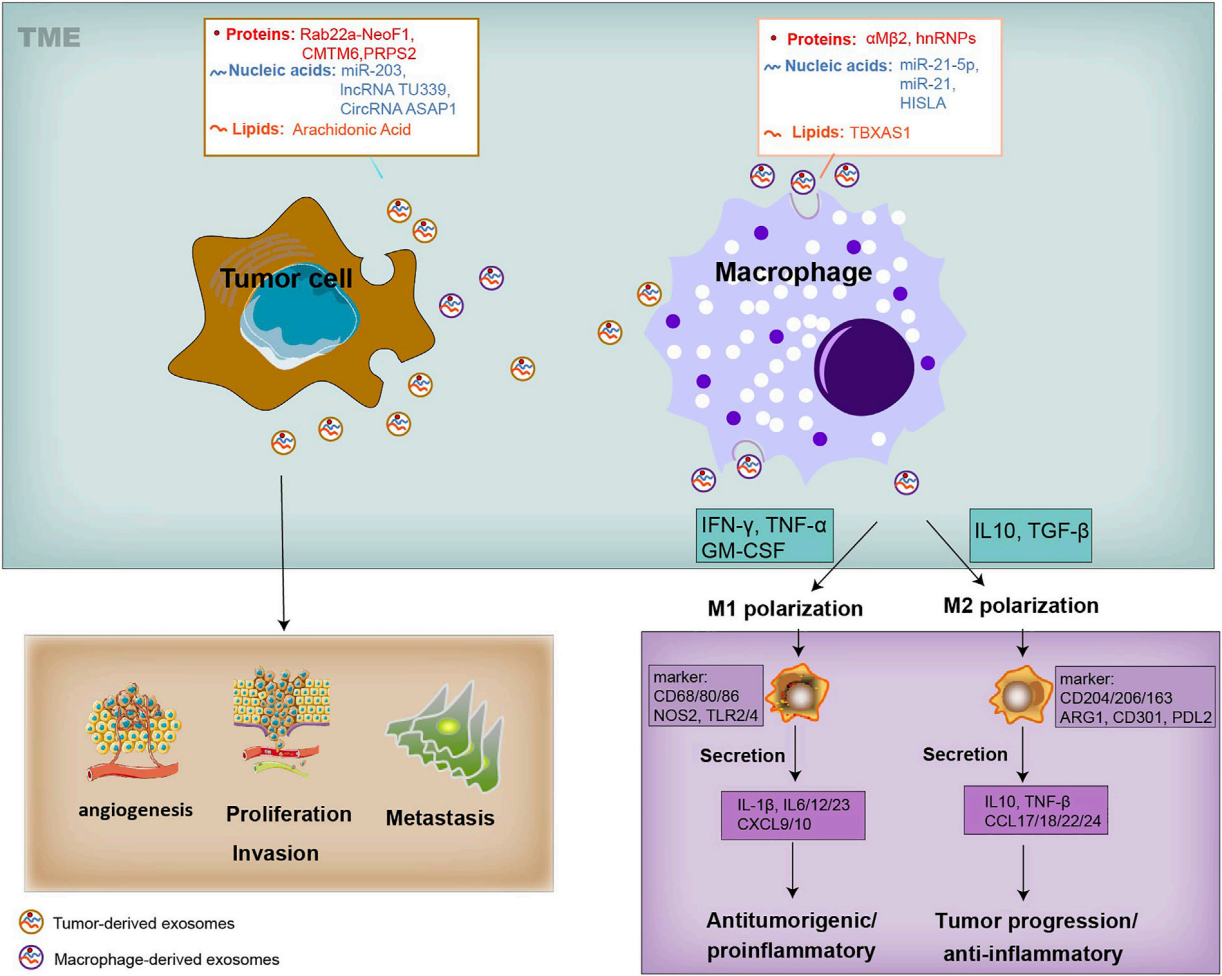
Systematic representation of the exosome-mediated crosstalk between TAMs and tumor within the TME. Exosomes can be released by TAMs and tumor cells, and transfer information to the recipient cells. Exosomes derived from TAMs can transfer their cargo proteins (α_M_β2, hnRNPs), nucleic acids (miR-21-5p, miR-21 and HISLA), and lipids (TBXAS1) to TME by tumor cells, affecting tumor biology, including cell proliferation, angiogenesis, metastasis, and T-cell cytotoxic activity. Tumor cells-derived exosomes cargo proteins (Rab22a-NeoF1, CMTM6, PRPS2), nucleic acids (miR-203, TU339 and circASAP1), and lipids (AA), and educate the polarization and differentiation of macrophages into the cancer-inhibiting M1 (proinflammatory) and cancer-promoting M2 phenotype (anti-inflammatory). The activation of M1 type macrophages occurs through signal transduction of IFN-γ, TNF and Toll-like receptors (TLR). M1 cells produce nitric oxide (NO), reactive oxygen species (ROS) and pro-inflammatory cytokines such as interleukin (IL)-1β, IL-6, IL-12, IL-23, CXCL9, CXCL10, TNF-α, and MHC molecules. Genetic markers related to M1 polarization include IL1a, IL1b, IL6, NOS2, TLR2, TLR4, CD80 and CD86. The activation of M2 macrophages is mediated by various cytokines. M2 cells tend to express an immune suppressive phenotype, and produce anti-inflammatory cytokines, such as IL-10, TGF-β, CCL17, CCL18, CCL22, and CCL24. Surface proteins include CD115, CD206, PPARG, ARG1, CD163, CD301, Dectin-1, PDL2 and Fizz1.

Both tumor cells and TAMs undergo changes in cellular metabolism, shaping their functional phenotype in a way of mutual influence. As we know, tumor cells along with various components within the TME, and complex crosstalk between them, are closely involved in tumor development and metastasis (Figure 1). It is generally believed that tumor cells secrete diverse exosomes that contain several types of molecules and transport these molecules through the blood or surrounding cells in the TME (Nabet. et al., 2017; Devhare and Ray, 2018). These mediators, unlike cytokines that are directly secreted by macrophages, are also contained in macrophage-derived exosomes and can also be transmitted to recipient tumor cells, thereby facilitating tumor biological functions, such as proliferation, invasion, vascularization, and exerting appreciable influence on the tumor development and metastasis (Figure 1). Similarly, tumor-derived exosomes alter the phenotype of macrophages, triggering a polarized M1 or M2 phenotype (Ham et al., 2018) (Figure 1). The polarization of macrophages, being associated with tumor progression, also provides cancer treatment strategies concentrating on macrophages to improve long-term survival. The M1 macrophages are activated through signal transduction and then encourage inflammation response against invading pathogens and tumor cells. In contrast, the M2 macrophages are activated by several cytokines, express an immune suppressive phenotype and produce anti-inflammatory cytokines, thereby contributing to tumor-related immune dysfunction and progression (Mantovani et al., 2002; Biswas et al., 2013; Shapouri-Moghaddam et al., 2018) (Figure 1). Furthermore, it is worth noting that the transform of M1 and M2 phenotypes is continuous, so macrophages in the middle stage can express some of the M1 or M2 markers at the same time. TAMs represent a promising and effective target for cancer therapy, and strategies have been proposed to suppress TAM recruitment, to deplete their number, to switch M2 TAMs into antitumor M1 phenotype and to inhibit TAM-associated molecules.

## Exosomes: Biogenesis, Composition, Function and Isolation

### Biogenesis

The biogenesis of EVs depends on the endocytic membranes for exosomes and local microdomains in the plasma membrane for ectosomes, which control the accumulation of proteins and various types of RNA associated with their cytosolic surface. The biogenesis processes can be observed in immune cells, mesenchymal stem cells, fibroblasts, endothelial cells (ECs) and epithelial cells. In addition to the difference in size, there are only partial differences in the assembly, composition and release of each type of EVs (Meldolesi, 2018). Unlike ectosomes, exosomes are formed firstly by the membrane invagination of early endosomes. The formed intracellular multivesicular bodies (MVBs) contain intraluminal vesicles, where their contents can be fused and degraded (Jurj et al., 2020), and can be released into the extracellular space (Figure 1). However, ectosomes released immediately after generation. A core component of exosome biogenesis involves the endosomal sorting complexes required for transport complexes (ESCRT complexes), which composed of ESCRT-0, -I, -II, and -III, and also there is an ESCRT-independent pathway involving ceramides. The multiprotein complexes participate in recruiting deubiquitinating enzymes and further sort proteins into intraluminal vesicles (ILVs) (Maia et al., 2018; Lan B. et al., 2019). In addition, argonautes (Agos) are important miRNA-processing proteins, but cells secrete Agos independently of exosomes. It has been demonstrated that miRNAs and argonautes remain hardly detectable in common isolates of EVs (Makarova et al., 2021). It is worth noting that Agos sorting miRNA in exosome are not associated with classical pattern displaying exosomal marker CD63/CD81/CD9 (Jeppesen et al., 2019). Multiple studies have reported that other RBPs like Ago2, human antigen R (HuR) (Gu et al., 2021), and hnRNPK (Xu et al., 2019) are present in exosomes with possible roles for sorting of RNA. It has been shown that Ago2 binds and sorting miRNA into EVs through the KRAS-MEK-ERK signaling pathway. The KRAS-MEK-ERK pathway-dependent phosphorylation of Ago2 has been shown to exert some specific control over the sorting of let-7a, miR-100, and miR-320a into exosomes (Arroyo et al., 2011; McKenzie et al., 2016).

### Composition

Proteins enriched in exosomes contain membrane trafficking proteins (Rab proteins, ARFs and Annexins), as well as other transmembrane proteins such as lysosomal associated membrane proteins (LAMPs) and TfR; also enriched are surface biomarkers include tetraspanins (CD9, CD63, CD81), ceramide, flotillin; major histocompatibility complex (MHC) molecules for antigen presentation; adhesion molecules such as integrins, ICAM1 and surface peptide (Steinbichler et al., 2019) (Figure 1). Some of these molecules, such as MHC I molecules and tetraspanins, are ubiquitously expressed, but their abundance varies in different cell types, activation status and community microenvironment (Leone et al., 2018). The nucleic acids in exosome isolates include mRNA, miRNA, long non-coding RNA (lncRNA), circRNA, mtRNA, transfer RNA (tRNA), snRNA, snoRNA, and piRNA (Maia et al., 2018; Meldolesi, 2018). The lipid component of exosomes comprises ceramide, cholesterol, phosphatidylserine, sphingomyelin, hexosylceramides, saturated fatty acids, and other surface proteoglycans. The membrane lipid lysobisphosphatidic acid, which is absent in other cellular membranes, leads to the accumulation of cholesterol (Colombo et al., 2014).

**FIGURE 2:**
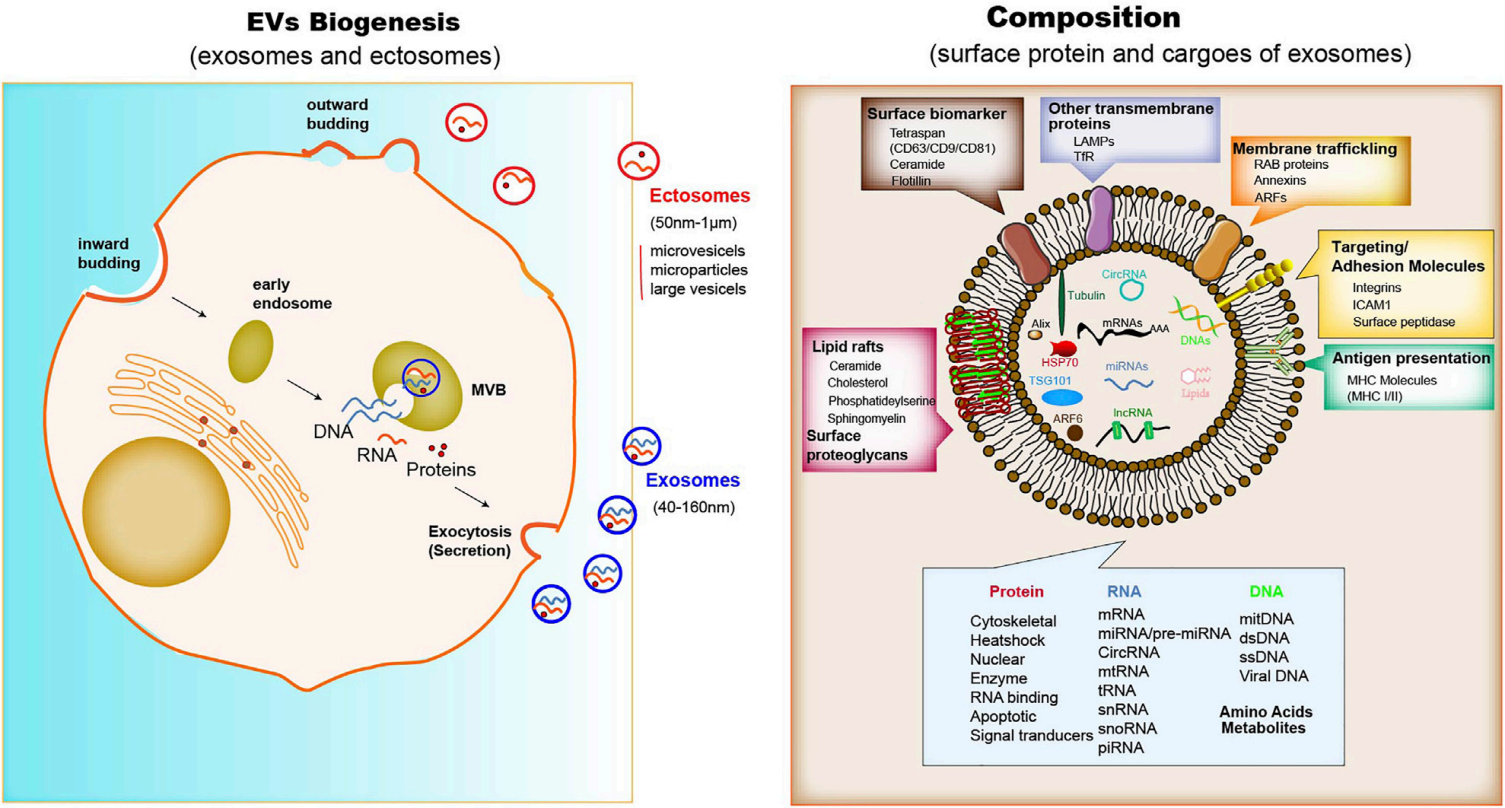
The Biogenesis, composition and function of exosomes. When MVBs fuse with the plasma membrane, exosomes are released (size range ∼40–160 nm), which were then endocytosed by phagocytic mechanism or the receptor-ligand interaction with the donor cell. The membrane of exosomes is enriched in tetraspanins, small transmembrane proteins that are crucial for trapping both membrane and luminal proteins. The common protein repertoire in exosomes derives mostly from the expression of endosomal markers (including surface biomarkers such as the tetraspan family (CD63/CD9/CD81), ceramide, and flotillin; other biomarkers such as Alix, ARF6 and TSG101), transmembrane proteins, MHC molecules, targeting and adhesion molecules (ICAM1, EpCAM), integrins, and surface peptidases. Nucleic acids comprise mRNAs, miRNAs, lncRNAs and cirRNAs, Lipid rafts contain ceramide, cholesterol, phosphatideylserine and sphingomyelin, and other surface proteoglycans. The adhesion protein ICAM-1 appears to be present only in the exosome membrane. The membrane of ectosomes is abundant in some receptors, glycoproteins and metalloproteinases. The cargoes trapped within the EVs contain many proteins and types of nucleic acids. Upon release, the two types of vesicle navigate through extracellular fluid for varying times and distances. Cargo proteins are: cytoskeletal and associated proteins, such as actin, vimentin, talin and annexin; a few chaperones, such as Hsp70 (a chaperone that protects the structure and function of proteins) and Hsc70; and a number of enzymes, such as phosphoglycerate kinase 1 (PGK1) and glyceraldehyde-3-phosphate dehydrogenase (GAPDH). Various GTPases, such as ARF6, are involved in the regulation of the ILV assembly processes.

Similar to exosomes, ectosome membranes have high levels of cholesterol, sphingomyelin, and ceramide, but low levels of tetraspanins and a few receptors. Some of these proteins, such as the matrix metalloproteinase MT1-MMP, two glycoprotein receptors (GP1b and GPIIb/GPIIa), the adhesion protein P-selectin, and the integrin Mac-1, are only present in the subpopulations of ectosomes (Colombo et al., 2014; Chen et al., 2016). For both exosomes and ectosomes, the surface and luminal cargoes are heterogeneous when comparing vesicles released by different cell types or by single cells in different functional states (Crescitelli et al., 2013). Together with luminal proteins, the ILVs, via mechanisms including a cooperation between ESCRTs and tetraspanins, accumulate RNAs, such as miRNAs, and proteins that modulate RNA function, such as RNA-binding proteins. non-coding RNAs and DNA sequences have also been found among cargoes of the exosome lumen (Klinge, 2018; Kumar et al., 2020) (Figure 2). In terms of specific cytosolic proteins, in ectosomes lumen it is similar to that in exosomes, most of which have cytoskeletal functions. The ESCRT mechanism drives several proteins that are active in ectosome assembly (Meldolesi, 2018). Other proteins directly interact with the plasma membrane. RNAs are also enriched within ectosomes mostly miRNAs, but also mRNAs and ncRNAs (Crescitelli et al., 2013).

### Function

It is worth noticing that exosomes released from the host cell surface are able to fuse with the plasma membranes of recipient cells and deliver their contents into the cytoplasm. Moreover, the contents of exosomes are determinant factors and are capable of TME modulation through stimulating cellular receptor signaling and regulating cell mortality (Sung et al., 2015). Exosomes derived from specific types of tumor cells and other cells surrounding the TME can facilitate tumor microenvironment by binding with receptors on the cell membrane, thereby facilitating tumor proliferation, invasion, metastasis, and chemoresistance. Therefore, functional molecules representing oncogenic signatures could cause TME alteration, expansion of the target recipient cells and activation of tumor-promoting signaling pathways. Such molecules contained in the exosomes are considered as diagnostic and prognostic biomarkers, and targets for cancer treatment (Lan B. et al., 2019). Due to the transfer and presentation of antigenic peptides, delivery of signals, gene-expression manipulation by exosomal miRNA, and induction of different signaling pathways by surface ligands present on the exosomes, the function of exosomes in immune regulation is specifically expressed as eliciting adaptive and innate immune reactions, supporting their application for therapy development and a potential role in regulating immune reactions in response to infectious agents or cancer (Kalluri and LeBleu, 2020).

### Isolation

#### Exosome Isolation Techniques

Techniques for exosome isolation have only been developed in the past few decades. For different purposes and applications, different isolation methods are selected, the techniques can be broadly classified based on their key mechanism: centrifugation techniques (ultracentrifugation (UC) and density gradient (DG) centrifugation), size-based isolation techniques (ultrafiltration and size exclusion chromatography), capture-based techniques, polymer precipitation, and microfluidics techniques (size-based microfluidics, immunoaffinity capture (IAC), and microfluidic separation)[]. The size similarity between exosomes and other EVs, which include ectosomes and MVs, has deeply impeded the development of isolation processes (Zhang et al., 2020; Zhu et al., 2020).

UC is currently the most widely used isolation technique. DG centrifugation is a derivative of UC, and is used to separate exosomes based on differences in size and density between the exosomes and other components, which usually require different centrifugation forces and times for pelleting. So it is considered the “gold standard” for exosome extraction and separation (Iwai et al., 2016). This method avoids cross-contamination, but it is time-consuming, costly, structurally damaged, easy to aggregate into clumps, which is not conducive to downstream analysis. Differential centrifugation and flow cytometry are the commonly used isolation and detection methods for microvesicles isolation (Zhao et al., 2021). Size-based isolation techniques mainly refer to ultrafiltration and size exclusion chromatography (SEC). Compared with UC, ultrafiltration techniques provide a higher particle yield, and also show the highest recovery of particles of less than 100 nm. The application of SEC is quick, easy, and low-cost. The isolated exosomes have complete structure and uniform size, and no significantly adversely effects on their biological characteristics, but they may be low purity due to the incorporation of other particles of similar size (Xu et al., 2015). Compared to ultracentrifugation, the IAC methodology, which involves specific immunoaffinity and magnetic bead capture mechanisms, is the most efficient technique for exosome enrichment with strong specificity, high sensitivity, high purity and high yield. It also can be used for qualitative and quantitative determination of exosomes. Nevertheless, the storage conditions of exosomes obtained by immunoaffinity chromatography are relatively harsh and are not suitable for large-scale separation of exosomes (Amrollahi et al., 2019). The method of polymer precipitation usually uses polyethylene glycol (PEG) as a medium to harvest exosomes under the centrifugation condition by reducing the solubility of the exosomes. Modified polymer co-precipitation (ExtraPEG) is more costly effective in terms of purity and recovery than ultracentrifugation and commercially available commercial kits (Yang X.-X. et al., 2019). Precipitation-based methods are the most attractive due to their simplicity and rapidity, no exosomal damage, and the low demand for equipment condition. However, it has been revealed that these methods are affected by the co-separation of various contaminants in the sample, including non-exosomal proteins and other particles. In addition, isolated exosomes might contain biopolymers that can further made sample analysis complicated (Zhu et al., 2020). Microfluidics-based techniques are known for their unique characteristics, including low cost and low time requirements. Microfluidics systems are ideal tools for separating exosomes from other nanometer-sized particles since they support cost-efficient, high-speed, and precise isolation processes. At present, the widespread use of microfluidics tools is fully integrated with size-based separation, immunoaffinity-based separation, and dynamic separation. Although it has numerous advantages such as high purity, controllability, isolation specificity, and high efficiency, there are still some problems, including the need for complicated equipments for isolation and the limitations of high immunoaffinity [57].

Although a variety of methods have been developed for the isolation and purification of exosomes, there exist specific advantages and disadvantages in terms of efficiency, reproducibility, and impact on functional outcomes. The combination of different isolation methods may be better than the single techniques. Unfortunately, these methods may not be able to completely obtain purified specific subpopulations, and probably obtain the EVs heterogeneous populations include exosomes (Kalluri and LeBleu, 2020). Furthermore, methods for exosome characterization are divided into two types: external characterization (mainly morphology and particle size detection) and inclusion characterization (like membrane protein, lipid raft). In general, the identification of isolated exosomes is recognized from three levels, including TEM identification of exosome morphology, NTA identification of exosome size and Western Blot identification of exosome surface protein markers (Yang X.-X. et al., 2019).

#### Exosomal RNA Methods

In the downstream analysis of exosomal content, a number of alternative exosomal RNA (exoRNA) extraction methods have been used, including phenol-based techniques (TRIzol) and combined phenol and pure column-based techniques (miRNeasy and HiPure Liquid RNA/miRNA kit (HLR)). In addition, commercial kits (SeraMir™ Exosomes RNA Amplification kit (SeraMir), Total Exosomes RNA and Protein Isolation kit (TER)) have been designed specifically for the isolation of RNA and protein from a single enriched exosome preparation. Moreover, the exoRNeasy Serum/Plasma kits isolate exoRNA directly from serum or plasma using a membrane-based affinity binding step. ExoRNA was further assessed using NanoDrop, Bioanalyzer 2100, quantitative polymerase chain reaction and RNA sequencing. It has been found that combinations of the TEI and TER methods resulted in high extraction efficiency and purity of small RNA obtained using CCM. ExoRNA isolated from serum by exoRNeasy showed high yield and narrow size distribution pattern of small RNA. In RNA profile analysis, the composition ratio of small RNAs, the content and quantity of miRNAs vary due to methodological differences (Tang et al., 2017).

## Tumor-Derived Exosomes in the Regulation of Macrophages Polarization

Many studies have attempted to illustrate a novel crosstalk between tumor and immune cells among TME. They have demonstrated that tumor-derived exosomes play a vital role in the conversion of monocyte-derived macrophages into regulatory macrophages and the mediation of cancer-related inflammation and tumor development (Lawrence and Natoli, 2011; Garzetti et al., 2014) through transferring their cargoes (Seo et al., 2018; Baig et al., 2020), including proteins, nucleic acids, and lipids, to recipient cells, such as macrophages in the TME, thus exhibiting antitumorigenic or tumorigenic effects (Figure 1; Table 1). Therefore, depletion of exosomal cargoes and interrupt the communication between tumor and macrophages can reverse some of the detrimental effects on tumor progression and restore drug sensitivity to chemotherapy.

**TABLE 1:** Tumor-derived exosomal cargoes stimulate polarization of macrophages.

Exosomal cargo	Cancer type	Recipients	Role/Mechanism	Major outcomes	References
Cytoskeleton-Centric Proteins	Colorectal cancer	Macrophages	Activate cathepsin B/pro-inflammatory cytokine secretion	Promote inflammation	Chen et al. (2016)
Rab22a-NeoF1	Osteosarcoma	Macrophages	M2 polarization/STAT3/RhoA activation	Promote lung metastasis	Zhong et al. (2021)
MFG-E8	Prostate cancer	Macrophages	M2 polarization/Increase efferocytosis/Suppress proinflammatory response	Promote tumor progression	Soki et al. (2014)
CMTM6	OSCC	Macrophages	M2 polarization/ERK1/2 signaling	Facilitate proliferation, migration and invasion	Pang et al. (2021)
HMGB1	Esophageal squamous cell carcinoma	Macrophages	M2 polarization/Trigger clonal expansion of PD1+ TAM	Promote tumor development	Li et al. (2019)
gp130	Breast Cancer	Macrophages	Activate IL-6/STAT3 signaling	Establish a pro-tumorigenic cancer microenvironment	Ham et al. (2018)
Chondroitin Sulfate Proteoglycan 4/α-2-Macroglobulin/Lactadherin/EGFR/Integrins	Glioblastoma multiforme	Monocytes	Increase phagocytic capacity/Mediate monocytes differentiation into M2 macrophages	Resemble the tumor supportive phenotypes	de Vrij et al. (2015)
EIF2/mTOR/Ephrin B	Glioblastoma	Macrophage	M2 phenotype/Increase PD-L1 expression	Induce immunosuppressive microenvironment	Gabrusiewicz et al. (2018)
Serglycin	Myeloma	Target cell	Reprogram target cells/Increase the migration of macrophage	Promote proliferation and invasion	Purushothaman et al. (2017)
PRPS2	NSCLC	Macrophages	M2 polarization	Promote DDP resistance	Liu et al. (2021)
miR-203	Colorectal cancer	Monocytes	M2 polarization/Promote the expression of M2 markers	Distant metastasis	Takano et al. (2017)
miR-145	Colorectal cancer cells	Macrophage-like cells	M2-like phenotype/Downregulate histone deacetylase 11	Modulate M2-like macrophage polarization and tumor progression	Shinohara et al. (2017)
miR-29a-3p	Oral squamous cell carcinoma	Macrophages	M2 polarization/Activate SOCS1/STAT6 signals	Promote tumor growth	Cai et al. (2019)
miR-301a-3p	Hypoxic pancreatic cancer cells	Macrophages	M2 polarization/Activate the PTEN/PI3K signaling pathway	Facilitate the invasion, migration and EMT; increase metastatic ability	Wang et al. (2018)
miR-155/miR-125b-2	Panc-1 cells	M1 macrophages	Trigger M1 to M2 polarization/increase M2 markers/decrease M1 markers	Enhance antigen presentation/T-cell activation/tumor destruction	(Su et al., 2016)
		M2 macrophages	Repolarize from M2 to M1/high iNOs/Arg1 and IL-1β/Arg1 ratio	Tumor invasion and metastasis	
miR-21/miR-155	Neuroblastoma	Unpolarized monocytes (prevalent M2)	Mixed polarization/exosomic mir-21/TLR8-NF-кB/exosomic mir-155/TERF1 signaling pathway	Development of drug resistance	Challagundla et al. (2015)
miR-146a-5p	Hepatocellular carcinoma	Macrophages	M2 polarization/activate NF-κB signaling/pro-inflammatory factors	Promote HCC progression	Yin et al. (2019)
miR-16	4T1 cells/Myeloma	Macrophages	Inhibit TAM infiltration and M2 polarization/IKKα suppression and I-κB accumulation	Suppress tumor growth	Jang et al. (2013); Khalife et al. (2019)
miR-125b-5p	Melanoma	Macrophages	TAM phenotype/target LIPA	Induce inflammation and angiogenesis; cell recruitment and survival	Gerloff et al. (2020)
miR-222-3p	Epithelial ovarian cancer	Macrophage	M2 polarization/Inhibit SOCS3/activate STAT3	Increase tumor size, microvessels, and lymphatic vessels	Ying et al. (2016)
miR-940	Ovarian Cancer	Macrophages	M2 polarization	Promote proliferation and migration	Chen et al. (2017)
miR-21-3p/miR-125b-5p/miR-181d-5p	Ovarian Cancer	Undifferentiated macrophages	M2 polarization/Suppress SOCS4/5/STAT3 pathway	Tumor progression	Chen et al. (2018)
miR-1246	Ovarian Cancer	Macrophages	M2 polarization/Target Cav1/p-gp	Confer chemoresistance and tumor progression	Kanlikilicer et al. (2018)
	GOF Mutp53 Colon Cancer Cells	Macrophages	M2 polarization/TGF-β activation	Promote cancer progression and metastasis	Cooks et al. (2018)
	Hypoxic glioma	Macrophage	M2 polarization/target TERF2IP/activate the STAT3 signaling/inhibit the NF-κB signaling	Formation of the immunosuppressive microenvironment	Qian et al. (2020)
miR-23a-3p	Tunicamycin treated HCC cells	Macrophages	Elevate PD-L1 in macrophages/inhibit T-cell function through PTEN-AKT pathway	Tumor cells escape from antitumor immunity	Liu et al. (2019)
miR-130/miR-33	Breast cancer	M2 macrophages/TAMs	Transform M2 to M1 phenotype	Inhibit tumor progression	Moradi-Chaleshtori et al. (2021b); Moradi-Chaleshtori et al., (2021a)
TU339	Hepatocellular carcinoma	Macrophages	M1/M2 polarization/regulate pro-inflammatory cytokine/co-stimulatory molecule expression/phagocytosis	Regulate cell proliferation	Li et al. (2018)
RPPH1	Colorectal cancer	Macrophages	M2 polarization/induce EMT; interact with TUBB3; prevent ubiquitination	Promote metastasis and proliferation	Liang et al. (2019)
BCRT1	MDA-MB-231	Macrophages	M2 polarization/modulate migration ability and chemotaxis of macrophages	Promote cell migration and angiogenesis	Liang et al. (2020)
SOX2-OT	NSCLC	Macrophages	miR-627-3p/Smad axis	Enhance the EGFR-TKI resistance	Zhou et al. (2021)
CircASAP1	Hepatocellular carcinoma	Macrophages	Tams infiltration/regulate miR-326/miR-532-5p-MAPK1 signaling/miR-326/miR-532-5p-CSF-1 pathway	Promote cell proliferation and invasion	Hu et al. (2019)
circ_00074854	HCC cell	Macrophages	Suppress M2 polarization	Inhibit migration and invasion	Wang et al. (2021)
circ_0044516	Prostate cancer	—	Downregulate miR-29a-3p expression	Promote proliferation and metastasis	Li et al. (2020)
circ-RanGAP1	Gastric cancer	—	miR-877-3p/VEGFA axis	Promotes progression	Lu et al. (2020)
Cdr1	Ovarian cancer	—	miR-1270/SCAI signaling pathway	sensitize ovarian cancer to cisplatin	Zhao et al. (2019)
circRNAs_102481	NSCLC	—	miRNA-30a-5p/ROR1 axis	Contribute to EGFR-TKIs resistance	Yang et al. (2021)
Arachidonic Acid	Aspc-1	Macrophages	M2-like phenotype/PGE2 secretion, increased secretion of pro-tumoral, bioactive molecules	Contribute to tumor progression	(Linton et al., 2018)

### Exosomal Protein

#### Macrophages M2 Polarization and Tumor Metastasis

It has been shown that some therapeutic fusion proteins can be taken up by surrounding cells to affect tumor progression. A new fusion protein Rab22a-NeoF1 can be sorted into exosomes with its binding partner PYK2, promotes M2 polarization to facilitate the pulmonary premetastatic niche formation and consequently promotes lung metastasis of recipient osteosarcoma cells. In detail, Rab22a-NeoF1 in the exosomes promotes cell migration and invasion through activation of RhoA by PYK2. In addition, the exosomal PYK2 induces the signal transducer and activator of transcription 3(STAT3)/RhoA activation in its recipient macrophages to increase M2 phenotype (Zhong et al., 2021). These findings provide a novel therapeutic strategy to interrupt metastasis by iRGD peptide to abolish the association of Rab22a-NeoF1 with its binding protein.

#### Macrophages M2 Polarization and Tumor Progression

Exosomes derived from tumor cells contain immunomodulatory and pro-angiogenic proteins, which can enhance immunosuppression and angiogenesis ability, thereby making recipient cells more aggressive and more prone to metastasis (Ekström et al., 2014). For example, exosomal cytoskeleton-centric proteins derived from CRC cells can be functionally transported to bone marrow-derived macrophages, and promote cancer-associated inflammation in exosome-educated macrophages through cathepsin B activity, pro-inflammatory cytokine secretion and the proportion of polarized cells (Chen et al., 2016). Coculture with apoptotic prostate cancer cells increases the expression of milk fat globule EGF factor 8 (MFG-E8) in macrophages, which of high expression in tissues and serum exosomes, finally suppresses the pro-inflammatory response and stimulates macrophage polarization into the activated M2 phenotype (Soki et al., 2014).

Additionally, the level of CKLF-like MARVEL transmembrane domain-containing 6 (CMTM6) is positively associated with the higher pathological stage of OSCC patients, CD163^+^ macrophage infiltration and PD-L1 expression. Exosomes from OSCC cells can deliver CMTM6 to macrophages and promote M2-like macrophage polarization to facilitate cells proliferative, migration and invasion through ERK1/2 signaling (Pang et al., 2021). High-mobility group box transcription factor 1 (HMGB1) containing exosomes can successfully trigger the clonal expansion of programmed cell death protein 1 positive (PD1^+^) TAMs, and finally facilitate esophageal squamous cell carcinoma (ESCC) development. Combining PD1^+^ TAMs targeting with exosomal HMGB1 would be an effective measure in immunotherapy (Li et al., 2019). Highly enriched of glycoprotein 130 (gp130) from breast cancer (BC)-derived exosomes triggers the secretion of interleukin-6 (IL-6) and activate the IL-6/STAT3 pathway (Ham et al., 2018). Furthermore, it has been found that serglycin exists in the exosomes derived from all human myeloma cell lines. Exosomal serglycin significantly promotes the proliferation and invasion of myeloma cells, and increases the migration of macrophage. In all, in myeloma-derived exosomes, serglycin and its binding partner are transferred to target cells, by which the recipient cells are reprogrammed to facilitate cancer progression (Purushothaman et al., 2017).

#### Macrophages M2 Polarization and Immunosuppression

In addition to polarization, tumor-derived exosomes can also mediate monocyte differentiation into M2 macrophages. For example, glioblastoma multiforme (GBM) EVs, including exosomes, can mediate the differentiation of peripheral blood-derived monocytes to alternative M2 phenotype macrophages. Several abundant proteins have been detected in these EVs, including chondroitin sulfate proteoglycan 4, α-2-macroglobulin, lactadherin, EGFR and integrins, and exposure to GBM EVs leads to modified expression of cell surface proteins, increases phagocytic capability and induces expression of M2 phenotype macrophage marker CD163 (de Vrij et al., 2015). Members of the STAT3 pathway are also present in glioblastoma (GBM)-derived stem cells (GSCs)-derived exosomes (GDEs), functionally mediating the immune-suppressive switch. In detail, GDEs pass through the cytoplasm of monocytes, leading to a reorganization of actin cytoskeleton, and leading the monocytes differentiation to the immunosuppressive M2 phenotype (Gabrusiewicz et al., 2018). Summarily, the same protein exerts different functions in the exosomes from different tumor cells, while exosomes secreted by the same tumor cell can transport multiple proteins to the recipient cell to initiate biological functions.

#### Macrophages M2 Polarization and Chemoresistance

It is reported that elevated phosphoribosyl pyrophosphate synthetases 2 (PRPS2) level is correlated with DDP resistance and poor prognosis in NSCLC patients. Downregulation of PRPS2 sensitizes DDP-resistant cells to DDP treatment. PRPS2 enriched in the exosomes mediates M2 macrophage polarization to promote DDP resistance of NSCLC cells (Liu G. et al., 2021).

#### Mixed M1/M2 Phenotype of Macrophages

Macrophages can be induced by exosomes to not only the M1 or M2 direction respectively, but also a “mixed” phenotype. For example, EVs derived from CRC cell lines and melanoma can both induce a mixed M1/M2 tumor-promoting macrophage phenotype (Bardi et al., 2018; Popēna et al., 2018). CRC cell lines released EVs can cause an increased surface biomarker CD14 expression and a mixed M1/M2 cytokine secretion pattern (C-X-C motif chemokine (CXCL) 10, IL-6, IL-23, and IL-10) in nonpolarized macrophages, and trigger IL-23 expression in M2 polarized macrophages (Popēna et al., 2018). Exosomes derived from melanoma also induce the expression of mixed phenotype markers including CC chemokine ligand (CCL)22, IL-12B, IL-1β, IL-6, i-NOS and TNF-α, which lead to M1 and M2 polarization (Bardi et al., 2018). Thus, the “mixed” phenotype is expected to contribute to multiple pro-tumor functions. However, the contents of these exosomes require further exploration.

### Exosomal miRNAs

#### Macrophages M2 Polarization and Tumor Growth and Metastasis

RNA sequencing analysis has demonstrated that miRNAs are of the highest content in microvesicle isolates derived from human plasma, accounting for over 42.32% of all raw reads and 76.20% of all mappable reads (Vlassov et al., 2012). In the early study, it was demonstrated that extracellular miRNAs were predominantly exosomes/microvesicles free. Cells in culture mainly export miRNAs in a form independent of exosomes. After ultracentrifugation at 110,000 g, most of the miRNAs in plasma and cell culture medium still remain in the supernatant, indicating that extracellular miRNAs are of non-vesicular origin (Wang et al., 2010; Turchinovich et al., 2011). Recent years, a large number of studies have shown that exosomal miRNAs play a crucial role in tumor progression, and stimulate angiogenesis and facilitate metastasis (Zhou et al., 2018; Zhang et al., 2019; Zhou et al., 2020). In addition, tumor-derived exosomal miRNAs polarize recipient macrophages and target diverse signaling pathways, leading to a positive or negative impact on tumor progression (Chen et al., 2018; Yin C. et al., 2019; Zhao et al., 2020).

Tumor-derived exosomal miRNAs promote cancer metastasis by regulating the crosstalk between cancer cells and TAMs, which also provide a therapeutic strategy for cancer therapy (listed in Table 1). For example, exosomes carrying miR-203 from CRC cells are incorporated into monocytes (Takano et al., 2017), while CRC cell-derived exosomal miR-145 (Shinohara et al., 2017) and exosomal miR-934 (Zhao et al., 2020) are uptaken by macrophages, which are then polarized into the M2 phenotype. It is demonstrated that package of miR-934 into exosomes is mediated by hnRNPA2B1. Interestingly, polarized M2 macrophages could induce premetasitatic niche formation and tumor metastasis by different signaling pathways. MiR-145 which is taken up by macrophage-like cells via EVs finally causes significant enlargement of the tumor volumes. In addition, OSCC-derived exosomal miR-29a-3p induces M2 polarization in macrophages and directly targets the suppressor of cytokine signaling (SOCS)1/STAT6 signaling to promote tumor growth (Cai et al., 2019). Exosome-enclosed miR-29a-3p promotes tumor growth in nude mice. In xenograft tumor tissues, the SCC-9-derived exosomes group exhibits highly expressed miR-29a-3p, lowly expressed SOCS1, and highly expressed p-STAT6. Furthermore, hypoxia induces the enrichment of released exosomes with various miRNAs from ovarian cancer, including miR-940 (Chen et al., 2017), miR-21-3p, miR-125b-5p, and miR-181d-5p (Chen et al., 2018), miR-222-3p (Ying et al., 2016), which can be transmitted to macrophages via exosomes and stimulate the M2 phenotype polarization, leading to the (SOCS)2/4/5/STAT3 cytokine signaling pathway suppression and tumor progression. The metastatic ability of pancreatic cancer cells is enhanced after being cocultured with macrophages or treated with hypoxic exosomes. Highly expressed miR-301a-3p in hypoxic pancreatic cancer cell-derived exosomes can be transferred to macrophages through the exosomes. Hypoxic exosomal miR-301a-3p is further demonstrated to stimulate macrophage polarization into the M2 phenotype in a HIF1a/2a-dependent manner, and facilitate malignant behaviors including cell invasion, migration and epithelial-mesenchymal transition (EMT) by activating the phosphatase and tensin homolog (PTEN)/phosphorinositol 3-kinase (PI3K) gamma signaling pathway *in vitro* and lung metastasis *in vivo* (Wang et al., 2018). Melanoma-derived miR-125b-5p is delivered to macrophages through exosomes and targets lysosomal acid lipase A (LIPA), which contributes to the formation of tumor-promoting TAM phenotype and promotes inflammation, angiogenesis and macrophage survival (Gerloff et al., 2020).

Exosomes secreted by colon cancer cells or glioma can deliver miR-1246 and induce a suppressive immune microenvironment. The miR-1246-enriched exosomes derived from TP53 mutants cancer cells are uptaken by macrophages and trigger them to reprogram into a tumor supportive and anti-inflammatory state via TGF-β activation (Cooks et al., 2018), and hypoxic glioma-derived exosomal miR-1246 induces M2 phenotype by targeting TERF2IP to activate the STAT3 signaling pathway and inhibit the NF-κB signaling pathway (Qian et al., 2020), subsequently promoting tumor proliferation, migration and invasion. Meanwhile, miR-23a-3p is one of the most abundant miRNAs in exosomes derived from tunicamycin (TM)-treated HCC cells (Exo-TMs). Treatment with Exo-TMs can promote the expression level of PD-L1 in macrophages *in vitro* and *in vivo*. The findings reveal that exosomal miR-23a-3p inhibits PTEN expression and elevates phosphorylated AKT, facilitating the formation of the immunosuppressive microenvironment (Liu et al., 2019). These results indicate that blocking cancer cell-macrophage communication mediated by exosomes may be a potential strategy for tumor treatment.

On the contrary, tumor-derived exosomal miRNAs exert negative effects on tumor progression. Hepatocellular carcinoma (HCC)-derived exosomes is found to reprogram macrophages by activating NF-κB signaling and inducing pro-inflammatory factors, and resulted in M2-polarized tumor-associated macrophages. Importantly, the transcription factor Sal-like protein-4 (SALL4) is a crucial regulator for miR-146a-5p, which is enriched in HCC exosomes. Blocking the SALL4/miR-146a-5p interaction in HCC reduced the expression of inhibitory receptors on T cells, reversed T cell exhaustion, and delayed HCC progression *in vivo* (Yin C. et al., 2019). Circulating miR-16 via exosomes in BC (Jang et al., 2013) and multiple myeloma polarizes macrophages towards a M2 phenotype and suppresses tumor growth (Khalife et al., 2019). Mechanically, exosomal miR-16 transferred to TAMs suppresses the I-κB kinase a (IKKa), leading to I-κB accumulation, and inhibits TAM infiltration and M2 polarization. Furthermore, loss of the miR-16 cluster supports polarization to M2 macrophages in a miR-15a-16-1-KO mouse model. Thus, cancer cell-derived exosomes induce immunosuppressive or pro-tumoral macrophages phenotype, suggesting further researches on the clinical application in immunotherapy.

#### Macrophages M2 Polarization and Chemoresistance

Exosomic miR-21/TLR8-NF-кB/exosomic miR-155/TERF1 signaling pathway is proved to facilitate the development of drug resistance. When non-polarized monocytes are co-cultured with neuroblastoma (NBL) cells, a mixed (but prevalently M2) polarization is induced, and the CD163+ cell count increases. Meanwhile, NBL cells cocultured with M1- and M2-polarized monocytes exhibit significant upregulation of miR-21/miR-155 and downregulation of TERF1, a telomerase inhibitor. Furthermore, monocyte-derived exosomal miR-155 is transferred to NBL cells and increases telomerase activity, while exosomal miR-21 can be transferred to monocytes by binding to toll-like receptor 8 (TLR8) in monocytes, and activating the NF-κB pathway. Xenografts injected with Dotap-miR-155 finally enhance tumor volumes *in vivo*. Downregulation of miR-21 in the xenografts decreases the number of CD163+ cells and downregulates the miR-155 levels. The results indicate that the exosomal miR-155/TERF1 axis plays a role in the miR-induced CDDP resistance in NBL cells, and these exosomes can be considered as therapeutic targets for drug resistance (Challagundla et al., 2015). Ovarian cancer cells transfer oncogenic miR-1246, which is abundantly expressed in OC exosomes, to M2 macrophages through exosomes, and inhibits Cav1 expression through PDGFβ receptors, thereby promoting cell proliferation. After transfection, the expression of miR-1246 in OC cells is almost 4 times higher than that of the scramble group. The combination of miR-1246 inhibitor treatment and chemotherapy sensitizes OC cells to paclitaxel and reduces the tumor burden *in vivo* (Kanlikilicer et al., 2018). These results indicate that after co-cultivation with tumor cells under different conditions, the non-polarized macrophages experience a switch of M1/M2 polarization status, leading to a switch in tumor chemoresistance.

#### Macrophages M1 Polarization and Tumor Inhibition

MiRNAs contained in tumor cell-derived exosomes also play a vital role in modulating the reprogram and M1 polarization of macrophages. For example, exosomes containing miR-130 or miR-33 are used to treat IL-4 induced M2 macrophages or TAMs, and the overexpression of miR-130 and miR-33 in exosomes increased the expression of M1 signature genes (IRF5, MCP1, CD80) and secretion of cytokines (IL-1β and TNF-α), thereby inhibiting tumor progression by transforming M2 to M1 phenotype through *in vitro* and *in vivo* analysis (Moradi-Chaleshtori et al., 2021b). Similarly, exosomes isolated from 4T1 BC cells are able to transfer miR-33 to M2 macrophages, and macrophages treated with 4T1-condition media are conversed from M2 to M1 phenotype as the expression of M1 markers is increased and the expression of M2 markers is decreased. Thus, exosomes can be used as an efficient nanocarrier for miR-33 delivery into macrophages, which is capable of inducing M1 polarization and suppressing tumor growth and metastasis (Moradi-Chaleshtori et al., 2021a).

TAM can also be reprogrammed through exosome cargo modification. For example, introducing miR-155 or miR-125b-2 plasmid DNA via a nanoparticles delivery system, which could be then stably expressed in exosomes, leads to macrophage reprogramming in the TME. Being cocultured with Panc-1 cells, M1 macrophages are enabled to polarize to M2 state due to the increased expression of M2 markers (Arg1) and decreased expression of M1 markers (IL-1β and iNOS), which could been shown the effect in cancer progression. Interestingly, after co-cultivation with Panc-1 cells transfected with miR-155 and miR-125b-2 plasmid DNA, the expression of Arg1 and iNOS decreases, and the expression of IL-1β increase slightly. The increase of IL-1β/Arg1 and iNOS/Arg1 ratio confirms that modified exosomes derived from plasmid DNA transfected Panc-1 cells facilitate the macrophage remolding from M2 phenotype back to M1 type, leading to suppression of tumor progression. Thus, the successful reprogramming of TAM plays a potential role in preventing tumor invasion and metastasis (Su et al., 2016).

### Exosomal lncRNAs

#### M2 Polarization and Tumor Progression

Emerging studies demonstrate that lncRNAs play a crucial regulatory role in tumorigenesis, macrophage activation and polarization via diverse signaling pathways (Han et al., 2019; Gao et al., 2020). The lncRNA-mediated crosstalk between TAMs and cancer cells contributes to tumor development. For instance, tumor-derived exosomal lncRNA TUC339 is in a complex interaction between tumor and macrophages through exosomes. THP-1 cells can absorb exosomal TUC339 to exert critical effects in M1/M2 macrophage activation, pro-inflammatory cytokine production regulation, co-stimulatory molecule expression, phagocytosis and cell viability (Li X. et al., 2018). Another lncRNA RPPH1 is transferred from CRC cell-derived exosomes into macrophages, mediates the M2 polarization of macrophages, increases the expression of vimentin and Ki67, and decreases the expression of E-cadherin, thereby facilitating metastasis and proliferation of cancer cells (Liang et al., 2019). In addition, lncRNA BCRT1 in MDA-MB-231 cell exosomes can be internalized by macrophages, significantly enhancing the expression of M2 phenotype markers (CD206 and MRC-2) and leading to M2 polarization; it also modulates the behaviors of macrophages, including migration ability and chemotaxis, thereby significantly promoting cell migration and angiogenesis (Liang et al., 2020). Summarily, exosomal lncRNAs from donor cells participate in various biological functions in recipient cells. Further investigations are still needed to discover other exosomal lncRNAs involved in macrophage polarization and tumor progression.

#### M2 Polarization and Chemoresistance

M2 macrophages and TAMs promote the epidermal growth factor receptor tyrosine kinase inhibitors (EGFR-TKIs) resistance in non-small cell lung cancer (NSCLC) cell lines. LncRNA SOX2 overlapping transcript (SOX2-OT) is highly expressed in NSCLC cells-derived exosomes. Transfer of SOX2-OT from NSCLC cells to macrophages through exosomes promotes macrophages M2 polarization and inhibit M1 polarization, which would enhance the EGFR-TKI resistance. Mechanically, SOX2-OT acts as miR-627-3p sponge to facilitate Smad2, Smad3 and Smad4 expression (Zhou et al., 2021).

### Exosomal circRNAs

#### M2 Polarization and Tumor Progression

A previous microarray assay demonstrated that 189 circRNAs were differentially expressed between M1 and M2 macrophages, functioning as tumor suppressors or oncogenes (Bach et al., 2019), and provided new directions on the role of circRNAs in the differentiation and polarization of macrophages (Zhang et al., 2017), and cell proliferation by sponging miRNAs (Hansen et al., 2013). For example, circASAP1 is considered as a competing endogenous RNA (ceRNA) of the suppressive miR-326 and miR-532-5p, directly targets MAPK1/CSF-1, and mediates TAMs infiltration by activating the miR-326/miR-532-5p-MAPK1/CSF-1 pathway (Hu et al., 2019). Macrophages incubated with HCC cell-derived exosomes containing lower level of circ_00074854 showed notably reduced level of IL-10 compared to those incubated with HCC cell-derived exosomes, indicating that exosomes with low circ_00074854 level could suppress macrophage M2 polarization, which in turn suppressing migration and invasion of HCC cells both *in vitro* and *in vivo* (Wang et al., 2021). Exosomal circ_0044516 (Li T. et al., 2020) and circ-RanGAP1 (Lu et al., 2020) promote the migration and invasion of cancer cells. Thus, these findings indicate that circRNAs polarize macrophages and affect tumor migration, invasion through exosomes. The communication mediated by exosomes between tumor and macrophages is still needs to be further explored.

#### Chemoresistance

A circRNA microarray analysis reveals that circRNAs_102481 are differentially expressed in the exosomes isolated before and after EGFR-TKIs resistance from NSCLC patients. Exosomal circRNA_102481 enhances cell proliferation and inhibits apoptosis. Importantly, tumor-derived exosomal circRNA_102481 could contribute to EGFR-TKIs resistance through the miRNA-30a-5p/ROR1 axis in NSCLC. In contrast, silence of circRNA_102481 and si-circRNA_102481 transferred by exosomes could finally decrease EGFR-TKIs resistance cell proliferation and induce cell apoptosis (Yang B. et al., 2021). In addition, exosomal Cdr1 can sensitize ovarian cancer to cisplatin through regulating the miR-1270/SCAI signaling pathway (Zhao Z. et al., 2019). Altogether, these results indicate that circRNAs affect tumor resistance through exosomes. The research on the circRNAs enveloped in tumor-derived exosomes is still at an early stage. Whether more circRNAs play a role in the TME modulation remains to be further investigated.

## Tumor-Associated Macrophages-Derived Exosomes Influence Tumor Progression

As crucial mediators of intercellular communication, exosomes released from TAMs also transfer their cargoes such as proteins, ncRNAs and lipids with the TME, conferring different phenotype to cancer cells, which are play vital roles in tumor initiation and metastasis. A proteomic analysis reveals that differential proteins, including cathepsins, 20S proteasome subunits, ribosomal proteins and heterogeneous nuclear ribonucleproteins (hnRNP), are found in exosomes released from TAMs, indicating that TAMs could release exosomal proteins with enhanced proteolytic activity and weakened RNA binding capacity. In addition, RNA proteins including 40S and 60S ribosomal proteins, HNRPAB, AGO2, NPM1 and NCL were differentially expressed between Ana-1 and TAM-derived exosomes, which may result in different RNA profiles in these exosomes (Zhu et al., 2015). Despite the roles of TAMs in immunomodulation and promotion of oncogenesis are well elucidated, TAMs-derived exosomal cargoes regulate tumor progression within the TME still need to be investigated.

### Exosomal Proteins

#### Tumor Migration

Studies have revealed that TAMs-derived exosomes function on tumor cells. A comparative analysis shows that many proteins which contained in EVs, including exosomes, released from TAMs are undetected in exosomes from macrophages. Some of the proteins can be existed both in colorectal tumors and mammary carcinomas. These proteins have crucial roles in the signal transduction, inflammation and immune response (Cianciaruso et al., 2019), and tumor progression (Lee et al., 2012). For instance, the integrin, αM β2 (CD11b/CD18), was also identified and notably specific and efficient in M2 macrophage-derived exosomes. Transfer of the CD11b/CD18 protein mediate by exosomes from TAMs to HCC cells can activate the MMP-9 signaling pathway and enhance cell’s migratory potency to support tumor migration (Wu et al., 2021). In addition, it has been shown that apolipoprotein E (ApoE) is mainly enriched in M2-polarized macrophages and M2-derived exosomes. M2 macrophage-derived exosomes mediate the intercellular transmission from M2 macrophages to gastric cancer (GC) cells, sustaining the connection between M2-exosomal ApoE, leading to the activation of the PI3K/Akt signaling pathway to remodel the cytoskeleton, and facilitating GC cells migration (Zheng et al., 2018). Therefore, these findings suggest that proteins transferred by TAM-exosomes mediate the attachment and detachment of target cells in the TME, thereby promoting the migration and invasion of cancer cells (listed in Table 2).

**TABLE 2:** Roles of macrophages-derived exosomal cargoes regulate tumor progression.

Exosomal cargo	Host cells	Recipient cells	Role/Mechanism	Major outcomes	References
Cathepsin/hnRNPs	TAMs	—	A proteomic analysis	Enhance proteolytic activity and decrease RNA binding capacity	Zhu et al. (2015)
Integrin α_M_β_2_	TAMs	HCC cells	MMP-9 signaling pathway	Support tumor migration	Wu et al. (2021)
ApoE	M2 macrophages	GC cells	Remodel the cytoskeleton-supporting migration/activate PI3K/AKT signaling	Promote GC migration	Zheng et al. (2018)
miR-142/miR-223	Macrophages	Hepatocellular carcinoma Cells	Decrease expression of reporter proteins and endogenously expressed stathmin-1 and insulin-like growth factor-1 receptor	Inhibit cell proliferation	Aucher et al. (2013)
miR-223	TAM	Breast Cancer cells	Mef2c-β-catenin pathway	Promote cell invasion	Yang et al. (2011)
miR-221-3p	TAM	OS cells	Activating the SOCS3/JAK2/STAT3 pathway	Aggravate the malignant behaviors	Liu et al. (2021)
miR-21-5p/miR-155-5p	Macrophages	Colorectal Cancer Cells	Target and bind to BRG1, decrease expression level of BRG1	Promote migration and invasion	Lan et al. (2019b)
miR-155-5p	TAM	Renal cell carcinoma	interacts with HuR and increases IFG1R mRNA stability	Enhance malignant phenotype	Gu et al. (2021)
	M2 Macrophage	Colon cancer cells	Impaire ZC3H12B-mediated IL-6 stability reduction	Induce immune escape and tumor formation	Ma et al. (2021)
miR-501-3p	M2 Macrophage	PDAC	TGFBR3-mediated TGF-β signaling pathway	Promote progression	Yin et al. (2019)
miR-155-5p/miR-221-5p	M2 Macrophage	endothelial cells	—	Promote angiogenesis	Yang et al. (2021)
miR-365	TAM	PDAC cells	Increases triphospho-nucleotide/Enhances the enzyme cytidine deaminase	Decrease sensitivity to gemcitabine	Binenbaum et al. (2018)
miR-223	TAM	EOC cells	miR-223/PTEN-PI3K/AKT signaling pathway	Chemotherapy resistance	Zhu et al. (2019)
miR-21	M2 macrophages	Gastric cancer cells	Downregulate PTEN/Activate PI3K/AKT signaling Pathway	Confer DDP Resistance	Zheng et al. (2017)
AFAP-AS1	M2 macrophages	Esophageal Cancer	Regulate miR-26a and ATF2 expression	Promote cell migration, invasion, and lung metastasis	Mi et al. (2020)
LIFR-AS1	Macrophages	Osteosarcoma cells	miR-29/NFIA axis	Promote tumor progression	Zhang et al. (2021b)
SBF2-AS1	M2 macrophage	Panc-1 cells	Regulate XIAP expression	Induce tumorigenic ability	Yin et al. (2020)
HISLA	TAMs	MDA-MB-231 cells	Block the interaction of PHD2 and HIF-1α to inhibit the hydroxylation and degradation of HIF-1α	Promote glycolysis and chemoresistance	Chen et al. (2019)
AGAP2-AS1	Macrophage	Radioresistant lung cancer cells	miR-296/NOTCH2 axis	Facilitate the malignant phenotypes	Zhang et al. (2021a)
COX1/TBXAS1	TAM	MC38	TXB2 production/Carry the biochemical machinery required for the synthesis of TXs	Promote cell migration	Cianciaruso et al. (2019)

### Exosomal miRNAs

#### Tumor Growth and Migration

TAMs infiltration is linked to osteosarcoma (OS) metastasis and growth. Overexpression of miR-221-3p enhances OS cell’s growth *in vitro* and *in vivo*, and exosomes enriched by miR-221-3p from TAMs aggravate the malignant behaviors of OS cells, such as proliferation, colony formation, migration and invasion by activating the SOCS3/JAK2/STAT3 pathway (Liu W. et al., 2021). In addition, high expression levels of miR-142 (Aucher et al., 2013), miR-223 (Yang et al., 2011), miR-501-3p, miR-21-5p and miR-155-5p can be transferred from macrophage to tumor cells through exosomes, thereby mediating the growth, migration and invasion of cancer cells. In detail, miR-21-5p and miR-155-5p directly bound to BRG1 and decrease its expression level, which is the main factor for M2 macrophage-derived exosomes induced CRC cell biology (Lan J. et al., 2019). Transfer of hypoxic TAM-derived miR-155-5p by exosomes also enhances malignant phenotype of renal cell carcinoma (RCC) by directly interacts with HuR and increases IFG1R mRNA stability. An *in vivo* analysis shows that miR-155-5p-deleted exosomes abrogates the hypoxic TAM-exosomes mediate tumor progression (Gu et al., 2021). Furthermore, both pancreatic ductal adenocarcinoma (PDAC) tissue and TAM-derived exosomes contain high expression level of miR-501-3p. Both M2 macrophage-derived exosomes and miR-501-3p promoted PDAC cell migration and invasion, as well as tumor formation and metastasis in nude mice. Inhibition of miR-501-3p levels in M2 macrophage exosomes can suppress the tumorigenic and metastasis ability, and also inhibit the expression of tumor cell stemness-related genes *in vivo*. It is worth noting that miR-501-3p derived from M2 macrophage suppresses the TGFBR3 gene by activating the TGF-β signaling pathway and contributes to the development of PDAC (Yin Z. et al., 2019).

#### Angiogenesis

It has been demonstrated that TAMs are beneficial for tumor angiogenesis. M2 macrophages are positively correlated with the microvessel density of PDAC tissues. Exosomes derived from M2 macrophages could promote the angiogenesis of mouse aortic endothelial cells (MAECs) *in vitro* and *in vivo*. In addition, RNA sequencing and qPCR analysis proved that miR-155-5p and miR-221-5p contained in the M2 macrophage-derived exosomes (MDEs), which could be transferred into MAECs (Yang Y. et al., 2021). These findings confirmed that the interaction between TAMs and the angiogenesis of cancer through exosomes, and targeting these exosomes would provide diagnostic and therapeutic strategy for cancer.

#### Chemoresistance

TAM-derived exosomal miR-365 increases triphospho-nucleotide and enhances the enzyme cytidine deaminase, thereby significantly increasing chemoresistance of PDAC cells to gemcitabine through the transfer of miR-365 in MDEs. Adoptive transfer of miR-365 in TAM induced gemcitabine resistance in PDAC-bearing mice, whereas immune transfer of the miR-365 antagonist recovered the sensitivity to gemcitabine (Binenbaum et al., 2018). Under hypoxic conditions, exosomes derived from macrophages are enriched in miR-223, which can be transferred to co-cultured EOC cells, resulting in an enhanced malignant phenotype and promoting the drug resistance of EOC cells through the PTEN-PI3K/AKT pathway *in vitro* and *in vivo* (Zhu et al., 2019). Studies have also demonstrated that MDEs display high levels of miRNA-21, which is found to confer DDP resistance in gastric cancer *in vitro* and *in vivo*. Exosomal transfer of TAM-derived miR-21 inhibits cell apoptosis and the expression of PTEN through the activation of PI3K/AKT signaling (Zheng et al., 2017). Briefly, these findings suggest that the exosomal cargoes may be a promising novel treatment target of chemoresistance in cancer patients.

#### Immune Escape

Evidence has highlighted that M2 macrophage regulation of cancer cells via exosome shuttling of miRNAs. Highly expressed miR-155-5p and interleukin (IL)-6 and poorly expressed ZC3H12B in MDEs. M2 macrophage-derived exosomal miR-155-5p promotes the development of colon cancer by inhibiting the expression of ZC3H12B *in vivo*. Delivery of miR-155-5p from M2 macrophage to colon cancer cells through exosomes impaires ZC3H12B-mediated IL-6 stability reduction, which consequently induced immune escape and tumor formation (Ma et al., 2021).

### Exosomal lncRNAs

#### Tumor Development

Similar to miRNA, multiple lncRNAs also participate in the crosstalk between the microenvironment and tumor cells directly and indirectly. The imbalanced level of lncRNAs from tumor cells may facilitate tumor initiation and development (Chen D. et al., 2019). For instance, lncRNA AFAP1-AS1 can be incorporated into M2 macrophage-derived exosomes and transferred to KYSE410 cells, and then downregulates miR-26a levels and upregulates the expression of ATF2, thereby promotes the invasion and *in vitro* lung metastasis of esophageal cancer (EC) (Mi et al., 2020). In xenograft nude mice model, MDEs promote osteosarcoma growth and metastasis *in vivo*. LncRNA LIFR-AS1 is highly expressed in macrophage-derived exosomes and promotes osteosarcoma cell proliferation, invasion via miR-29a/NFIA axis (Zhang H. et al., 2021). Moreover, high levels of lncRNA SBF2-AS1 in M2 macrophage-secreted exosomes also induces tumorigenic ability of PANC-1 cells (Yin et al., 2020). Thus, macrophages and the lncRNAs/target genes axis may provide novel targets for cancer therapy.

#### Chemoresistance

TAMs transmit a myeloid-specific lncRNA, HIF-1α-stabilizing long noncoding RNA (HISLA) through EVs to promote the aerobic glycolysis and chemoresistance of BC cells. In detail, HISLA destroys the interaction of PHD2 and HIF-1α, thereby suppressing the hydroxylation and degradation of HIF-1α. Interestingly, lactate released from glycolytic tumor cells increases the levels of HISLA in macrophages (Chen F. et al., 2019). Further researches are advised to better understand the functions of lncRNAs in modulating the TME and tumor progression, especially the crosstalk between macrophage-derived exosomal cargoes and cancer cells in the TME, which may be used for early diagnosis, treatment responses and targeted therapy.

#### Radiotherapy Immunity

Previous study has shown that exosomal lncRNA AGAP2 antisense RNA 1 (AGAP2-AS1) serves as a diagnostic biomarker of NSCLC. Downregulation of AGAP2-AS1 suppresses the tumor volume and weight of xenografts. Interestingly, exosomes overexpressing AGAP2-AS1(Oe-AGAP2-AS1-exo) and inhibiting miR-296 enhances tumor volume and weight of xenografts. Totally, inhibition of AGAP2-AS1, macrophage-derived exosomes, and exosomes overexpressing AGAP2-AS1 or miR-296 facilitates the malignant phenotypes of radioresistant lung cancer cells. Mechanically, AGAP2-AS1 negatively regulates miR-296, and NOTCH2 is targeted by miR-296. Thus, M2 macrophage-derived exosomal AGAP2-AS1 enhances radiotherapy immunity in lung cancer by reducing miR-296 and elevating NOTCH2 (Zhang F. et al., 2021).

### Exosomal Lipids

The biogenesis of EVs relies partially on various lipid pathways. Multiple lipid molecules or related enzymes, which regulate inflammation and tumor development, are transferred by EVs from their parent cells (Record et al., 2018). It is found that COX1 and TX A synthase 1 (TBXAS1) are significantly enriched in TAM-EVs and almost undetectable in MC38-EVs; the MC38 cells transfected with TAM-EVs are induced of TXB2 production, indicating that TAM-derived EVs transmit the biochemical molecule convertor necessary to TXs synthesis (Cianciaruso et al., 2019). In all, these results confirm that the exosome-mediated transfer of functional lipids from macrophages to tumor cells ultimately contributes to the pro-tumor activities of cancer cells.

## Exosomes as Biomarkers

As discussed above, exosomes extracted from several types of tumors or macrophages are enriched in proteins, nucleotides, and lipids, which are delivered into recipient cells through different media and can induce tumor-promoting phenotype of the recipient cells. These exosomes and their cargoes could be considered as biomarkers for the diagnosis and prognosis of cancers (Table 3). For example, exosomal MIF derived from PDAC establishes a pre-metastatic niche for fibrotic liver and enhances metastatic burden (Costa-Silva et al., 2015). Circulating exosomal miR-221-3p levels are closely associated with LN metastasis in cervical squamous cell carcinoma (CSCC) patients (Zhou et al., 2019). Moreover, exosomal miR-25-3p and miR-92a-3p released from liposarcoma cells (Casadei et al., 2017) and serum exosomal miR-203 in CRC (Takano et al., 2017) may also serve as biomarkers. In detail, the expression of miR-25-3p and miR-92a-3p are significantly higher in LPS cell-EVs with respect to EVs derived from normal preadipocytes, and the ROC curve supports their correlation with tumor diagnosis. High expression of serum exosomal miR-203 was associated with distant metastasis and an independent poor prognostic factor. In addition, exosomal lncRNA CRNDE-h is increased in CRC and positively correlated with lymph node metastasis, distant metastasis and poor survival rates (Liu et al., 2016). Thus, it has been attracting increasing attention to further understand the basic characteristics of and the crosstalk mediated by tumor- or macrophage-derived exosomes that leads to cancer progression and drug resistance during cancer chemotherapy and immunotherapy, and their potential clinical application.

**TABLE 3:** Clinical applications for exosomes in different types of cancer.

Exosomes application	Cargoes	Derived cells	Major outcome	References
Biomarkers	MIF	PDAC	Establish a pre-metastatic niche; enhance metastatic burden	Costa-Silva et al. (2015)
	miR-221-3p	CSCC	The levels of miRNA are closely associated LN metastasis	Zhou et al. (2019)
	miR-25-3p/miR-92a-3p	LPS	Correlation with tumor diagnosis	Casadei et al. (2017)
	miR-203	CRC	Correlated to distant metastasis and can be an independent poor prognostic factor	Takano et al. (2017)
	CRNDE-h	CRC	Correlate with lymph node metastasis and distant metastasis; poor survival rates	Liu et al. (2016)
Drug Delivery	Drugs/siRNAs	Ovarian cancer	Reverse immunosuppression caused by M2-TAMs	Kanlikilicer et al. (2018)
	Drugs (DOX and PTX)	Macrophage	Display anticancer ability in cancer metastasis	Batrakova and Kim, (2015); Kim et al. (2018)
Immunotherapy	Nanovesicles (M1NVs)	M1 Macrophages	Suppress tumor growth; promote antitumor efficacy of the checkpoint inhibitor therapy	Choo et al. (2018)
	Modified exosomes (Ce6-R-Exo)	Pancreatic Cancer	Generate reactive oxygen species; Release cytokines	Jang et al. (2021)
	Modified exosomes (siGRP78)	BM-MSCs	Enhance chemosensitivity to sorafenib	Li et al. (2018a)
	peptide antigens-based vaccine	DC	Stimulate CD4^+^ helper T cells and CD8^+^ CLTs to participate in the anti-tumor response	Hsu et al. (2003); Fu et al. (2020)
	T cells-based vaccine	T cells	Enhance CD4^+^ T-cell responses for trastuzumab-resistant HER2+ breast cancer	Xie et al. (2018)

## Exosome-Based Cancer Immunotherapy

Exosome-based therapies are emerging, cutting-edge strategies to suppress tumor progression or enhance anti-tumor immunity. Studies have demonstrated that exosomes can easily cross the biological barriers, such as the blood-brain barrier, and can be modified for stronger efficiency in recipient cancer cells. Due to their transfer ability, lipid bilayer structure and unique surface proteins, exosomes can be used as nanoparticle carriers for drugs, nucleic acids, and proteins to recipient cancer cells.

Immunotherapy is the treatment to induce immunity or promote the resistance of immune system to diseases including cancer, which is based on the innate and mostly on the adaptive immune system. Immune cells are stimulated by antibodies, other immune cells and genetic modifications for therapeutic purpose. Given the crucial role played by macrophages in EVs-mediated crosstalk, modulating macrophages is an effective strategy in tumor therapy. The inhibitory effects mediated by M1-like macrophages, therefore, can be applied in cancer therapy (Su et al., 2016) (Table 3). Exosomes are applied as the carriers for both drugs and siRNA to reverse immunosuppression caused by M2-TAMs (Kanlikilicer et al., 2018). In addition, it is another strategy to reprogram TAMs towards M1 phenotype. Treatment with exosome-mimetic nanovesicles derived from M1 macrophages (M1NVs) stimulates polarization of M2 macrophages to M1 phenotype *in vitro* and *in vivo*, and intravenous injection of M1NVs suppressed tumor growth *in vivo*; the antitumor effect is further enhanced after injection of a combination of M1NVs and PD-L1, suggesting that M1NVs repolarize M2 to M1 macrophages and promote antitumor efficacy of the checkpoint inhibitor therapy (Choo et al., 2018).

Tumor-derived modified exosomes loaded with chlorin e6 photosensitizer (Ce6-R-Exo) can be visualized by photoacoustic imaging and can efficiently generate reactive oxygen species in tumor cells under laser irradiation. They also increase the release of cytokines, indicating that the R-Exos can be used as drug carriers and immunotherapeutic agents (Jang et al., 2021). Exosomes contain tumor antigens such as MHC I and can be used as vaccines in cancer immunotherapy. DC-derived exosomes can be loaded with several peptide antigens (e.g., MHC I, MHC II) in the presence of APC, thereby stimulating CD4^+^ helper T cells and CD8^+^ CLTs to induce the anti-tumor response (Hsu et al., 2003; Fu et al., 2020). Subcutaneous injection of TAE-DC vaccines *in vivo* significantly restores the activated T cells in the TME and improves the therapeutic effect (Xiao et al., 2017). Moreover, HER2-specific exosome- and T cell-based vaccine can effectively enhance CD4^+^ T-cell responses, which provides a novel alternative for trastuzumab-resistant HER2+ BC therapy (Xie et al., 2018). EVs can stimulate both CD8^+^ cytotoxic T-lymphocytes and CD4^+^ T-helper cells, which are crucial for triggering an efficient anti-tumor immune response (Markov et al., 2019). Thus, modified macrophage- or tumor-derived exosomes can be used as cancer vaccines, providing important strategies for immune recognition and therapeutic intervention, and the delivery route, dosage and content of exosomes can be adjusted according to different means of immunotherapy (Table 3).

Current treatment strategies mainly focus on suppressing the production of cancer cell derived-exosomes and preventing the uptake of specific exosomes by target cells. GW4869, an inhibitor to exosome secretion and neutral sphingomyelinase, can inhibit the production of exosomes and achieve anti-tumor effects (Faict et al., 2018). Exosomes loaded with chemotherapeutic drugs such as doxorubicin and paclitaxel, can be used as a novel type of nano-preparation, displaying a highly effective anticancer ability in cancer metastasis model mice (Batrakova and Kim, 2015; Kim et al., 2018). Exosomes appear as potential carriers for therapeutic RNAs, including siRNA and miRNAs, to target cancer cells. Upon internalization by tumor cells, exosomes released by human mesenchymal stem cells (MSCs) act as prodrugs and induce the death of tumor cells, representing a novel therapeutic method for tumor. For instance, bone marrow mesenchymal stem cells (BM-MSCs) are modified to express the exosomal siGRP78. The modified BM-MSCs derived exosomes are able to deliver GRP78 siRNA to HCCs and then target GRP78, enhancing chemosensitivity to sorafenib in drug-resistant HCC and suppressing cell proliferation and invasion (Li H. et al., 2018). MSCs engineered to secret miR-379 encapsulated in EVs display exciting potency as an innovative therapy strategy for metastatic BC (O'Brien et al., 2018). Taken together, MSC-derived exosomes are considered as drug/nucleic acid delivery media; effective technologies are still needed to customize appropriate drug-loading capacity, increase the targeting efficacy of exosomes and reduce cytotoxicity.

## Conclusion and Perspectives

Exosomes and their cargoes play a crucial role in mediating crosstalk between tumors and TAMs, and reprograming the host immune response. TAMs associate with immune escape and express cytokines, chemokines that can suppress antitumor immunity and promote tumor progression (Wu et al., 2020). Therefore, targeting exosomes and suppressing their detrimental effects on macrophages opens a new view for the development of active anti-tumor agents, and reprogramming TAMs toward the M1 phenotype through modifying exosome cargoes can serve as a promising strategy to suppress tumor growth (Choo et al., 2018). In this review, we summarize how macrophages are induced to become immunosuppressive and how tumor cells are affected by macrophages, which may be applied in the further therapeutic development and clinical trials.

Although the significant functions of exosomes are revealed in cell co-culture systems, they still need to be further confirmed *in vivo* experiments. In addition, whether exosomes can growth or divide, and whether they can participate in signaling events and autonomous biochemical reactions under certain environment, these questions remain to be further determined (Kalluri and LeBleu, 2020). Despite of substantial achievements in the field, the communication network of exosomes in organisms is still far from being fully understood. In addition to the need for more information on basic issues, including biogenesis and the delivery mechanisms of exosomal cargoes, further steps are still necessary to standardize the purification, characterization and isolation of exosomes. There is still so much work to do before overcoming the barriers presently impeding exosome-based immunotherapeutic strategies for cancer.

## References

[B1] Amrollahi P. Rodrigues M. Lyon C. J. Goel A. Han H. Hu T. Y. (2019). Ultra-Sensitive Automated Profiling of EpCAM Expression on Tumor-Derived Extracellular Vesicles. Front. Genet. 10, 1273. 10.3389/fgene.2019.01273 31921310 PMC6928048

[B2] Arroyo J. D. Chevillet J. R. Kroh E. M. Ruf I. K. Pritchard C. C. Gibson D. F. (2011). Argonaute2 Complexes Carry a Population of Circulating microRNAs Independent of Vesicles in Human Plasma. Proc. Natl. Acad. Sci. 108 (12), 5003–5008. 10.1073/pnas.1019055108 21383194 PMC3064324

[B3] Aucher A. Rudnicka D. Davis D. M. (2013). MicroRNAs Transfer from Human Macrophages to Hepato-Carcinoma Cells and Inhibit Proliferation. J.I. 191 (12), 6250–6260. 10.4049/jimmunol.1301728 PMC3858238 24227773

[B4] Bach D.-H. Lee S. K. Sood A. K. (2019). Circular RNAs in Cancer. Mol. Ther. - Nucleic Acids 16, 118–129. 10.1016/j.omtn.2019.02.005 30861414 PMC6411617

[B5] Baig M. S. Rajpoot S. Liu D. Savai R. Banerjee S. (2020). Tumor-derived Exosomes in the Regulation of Macrophage Polarization. Inflamm. Res. 69 (5), 435–451. 10.1007/s00011-020-01318-0 32162012

[B6] Bardi G. T. Smith M. A. Hood J. L. (2018). Melanoma Exosomes Promote Mixed M1 and M2 Macrophage Polarization. Cytokine 105, 63–72. 10.1016/j.cyto.2018.02.002 29459345 PMC5857255

[B7] Batrakova E. V. Kim M. S. (2015). Using Exosomes, Naturally-Equipped Nanocarriers, for Drug Delivery. J. Controlled Release 219, 396–405. 10.1016/j.jconrel.2015.07.030 PMC4656109 26241750

[B8] Binenbaum Y. Fridman E. Yaari Z. Milman N. Schroeder A. Ben David G. (2018). Transfer of miRNA in Macrophage-Derived Exosomes Induces Drug Resistance in Pancreatic Adenocarcinoma. Cancer Res. 78 (18), 5287–5299. 10.1158/0008-5472.CAN-18-0124 30042153

[B9] Biswas S. K. Allavena P. Mantovani A. (2013). Tumor-associated Macrophages: Functional Diversity, Clinical Significance, and Open Questions. Semin. Immunopathol 35 (5), 585–600. 10.1007/s00281-013-0367-7 23657835

[B10] Cai J. Qiao B. Gao N. Lin N. He W. (2019). Oral Squamous Cell Carcinoma-Derived Exosomes Promote M2 Subtype Macrophage Polarization Mediated by Exosome-Enclosed miR-29a-3p. Am. J. Physiology-Cell PhysiologyCell Physiol. 316 (5), C731–C740. 10.1152/ajpcell.00366.2018 30811223

[B11] Casadei L. Calore F. Creighton C. J. Guescini M. Batte K. Iwenofu O. H. (2017). Exosome-Derived miR-25-3p and miR-92a-3p Stimulate Liposarcoma Progression. Cancer Res. 77 (14), 3846–3856. 10.1158/0008-5472.can-16-2984 28588009 PMC6033276

[B12] Challagundla K. B. Wise P. M. Neviani P. Chava H. Murtadha M. Xu T. (2015). Exosome-mediated Transfer of microRNAs within the Tumor Microenvironment and Neuroblastoma Resistance to Chemotherapy. J. Natl. Cancer Inst. 107 (7). 10.1093/jnci/djv135 PMC4651042 25972604

[B13] Chen D. Lu T. Tan J. Li H. Wang Q. Wei L. (2019a). Long Non-coding RNAs as Communicators and Mediators between the Tumor Microenvironment and Cancer Cells. Front. Oncol. 9, 739. 10.3389/fonc.2019.00739 31448238 PMC6691164

[B14] Chen F. Chen J. Yang L. Liu J. Zhang X. Zhang Y. (2019b). Extracellular Vesicle-Packaged HIF-1α-Stabilizing lncRNA from Tumour-Associated Macrophages Regulates Aerobic Glycolysis of Breast Cancer Cells. Nat. Cel Biol 21 (4), 498–510. 10.1038/s41556-019-0299-0 30936474

[B15] Chen X. Ying X. Wang X. Wu X. Zhu Q. Wang X. (2017). Exosomes Derived from Hypoxic Epithelial Ovarian Cancer Deliver microRNA-940 to Induce Macrophage M2 Polarization. Oncol. Rep. 38 (1), 522–528. 10.3892/or.2017.5697 28586039

[B16] Chen X. Zhou J. Li X. Wang X. Lin Y. Wang X. (2018). Exosomes Derived from Hypoxic Epithelial Ovarian Cancer Cells Deliver microRNAs to Macrophages and Elicit a Tumor-Promoted Phenotype. Cancer Lett. 435, 80–91. 10.1016/j.canlet.2018.08.001 30098399

[B17] Chen Z. Yang L. Cui Y. Zhou Y. Yin X. Guo J. (2016). Cytoskeleton-centric Protein Transportation by Exosomes Transforms Tumor-Favorable Macrophages. Oncotarget 7 (41), 67387–67402. 10.18632/oncotarget.11794 27602764 PMC5341883

[B18] Choo Y. W. Kang M. Kim H. Y. Han J. Kang S. Lee J.-R. (2018). M1 Macrophage-Derived Nanovesicles Potentiate the Anticancer Efficacy of Immune Checkpoint Inhibitors. ACS Nano 12 (9), 8977–8993. 10.1021/acsnano.8b02446 30133260

[B19] Cianciaruso C. Beltraminelli T. Duval F. Nassiri S. Hamelin R. Mozes A. (2019). Molecular Profiling and Functional Analysis of Macrophage-Derived Tumor Extracellular Vesicles. Cel Rep. 27 (10), 3062–3080. 10.1016/j.celrep.2019.05.008 PMC6581796 31167148

[B20] Colombo M. Raposo G. Théry C. (2014). Biogenesis, Secretion, and Intercellular Interactions of Exosomes and Other Extracellular Vesicles. Annu. Rev. Cel Dev. Biol. 30, 255–289. 10.1146/annurev-cellbio-101512-122326 25288114

[B21] Cooks T. Pateras I. S. Jenkins L. M. Patel K. M. Robles A. I. Morris J. (2018). Mutant P53 Cancers Reprogram Macrophages to Tumor Supporting Macrophages via Exosomal miR-1246. Nat. Commun. 9 (1), 771. 10.1038/s41467-018-03224-w 29472616 PMC5823939

[B22] Cortés M. Sanchez‐Moral L. de Barrios O. Fernández‐Aceñero M. J. Martínez‐Campanario M. Esteve‐Codina A. (2017). Tumor‐associated Macrophages (TAMs) Depend on ZEB1 for Their Cancer‐promoting Roles. EMBO J. 36 (22), 3336–3355. 10.15252/embj.201797345 29038174 PMC5686549

[B23] Costa-Silva B. Aiello N. M. Ocean A. J. Singh S. Zhang H. Thakur B. K. (2015). Pancreatic Cancer Exosomes Initiate Pre-metastatic Niche Formation in the Liver. Nat. Cel Biol 17 (6), 816–826. 10.1038/ncb3169 PMC5769922 25985394

[B24] Crescitelli R. Lässer C. Szabó T. G. Kittel A. Eldh M. Dianzani I. (2013). Distinct RNA Profiles in Subpopulations of Extracellular Vesicles: Apoptotic Bodies, Microvesicles and Exosomes. J. Extracellular Vesicles 2, 20677. 10.3402/jev.v2i0.20677 PMC3823106 24223256

[B25] de Vrij J. Maas S. L. N. Kwappenberg K. M. C. Schnoor R. Kleijn A. Dekker L. (2015). Glioblastoma-derived Extracellular Vesicles Modify the Phenotype of Monocytic Cells. Int. J. Cancer 137 (7), 1630–1642. 10.1002/ijc.29521 25802036

[B26] Devhare P. B. Ray R. B. (2018). Extracellular Vesicles: Novel Mediator for Cell to Cell Communications in Liver Pathogenesis. Mol. Aspects Med. 60, 115–122. 10.1016/j.mam.2017.11.001 29122679 PMC5856598

[B27] Ekström E. J. Bergenfelz C. von Bülow V. Serifler F. Carlemalm E. Jönsson G. (2014). WNT5A Induces Release of Exosomes Containing Pro-angiogenic and Immunosuppressive Factors from Malignant Melanoma Cells. Mol. Cancer 13, 88. 10.1186/1476-4598-13-88 24766647 PMC4022450

[B28] Faict S. Muller J. De Veirman K. De Bruyne E. Maes K. Vrancken L. (2018). Exosomes Play a Role in Multiple Myeloma Bone Disease and Tumor Development by Targeting Osteoclasts and Osteoblasts. Blood Cancer J. 8 (11), 105. 10.1038/s41408-018-0139-7 30409995 PMC6224554

[B29] Fu C. Zhou L. Mi Q.-S. Jiang A. (2020). DC-based Vaccines for Cancer Immunotherapy. Vaccines, 8, 706. 10.3390/vaccines8040706 PMC7712957 33255895

[B30] Gabrusiewicz K. Li X. Wei J. Hashimoto Y. Marisetty A. L. Ott M. (2018). Glioblastoma Stem Cell-Derived Exosomes Induce M2 Macrophages and PD-L1 Expression on Human Monocytes. Oncoimmunology 7 (4), e1412909. 10.1080/2162402x.2017.1412909 29632728 PMC5889290

[B31] Gao Y. Fang P. Li W.-J. Zhang J. Wang G.-P. Jiang D.-F. (2020). LncRNA NEAT1 Sponges miR-214 to Regulate M2 Macrophage Polarization by Regulation of B7-H3 in Multiple Myeloma. Mol. Immunol. 117, 20–28. 10.1016/j.molimm.2019.10.026 31731055

[B32] Garzetti L. Menon R. Finardi A. Bergami A. Sica A. Martino G. (2014). Activated Macrophages Release Microvesicles Containing Polarized M1 or M2 mRNAs. J. Leukoc. Biol. 95 (5), 817–825. 10.1189/jlb.0913485 24379213

[B33] Gerloff D. Lützkendorf J. Moritz R. K. C. Wersig T. Mäder K. Müller L. P. (2020). Melanoma-Derived Exosomal miR-125b-5p Educates Tumor Associated Macrophages (TAMs) by Targeting Lysosomal Acid Lipase A (LIPA). Cancers 12 (2), 464. 10.3390/cancers12020464 PMC7072270 32079286

[B34] Gu W. Gong L. Wu X. Yao X. (2021). Hypoxic TAM-Derived Exosomal miR-155-5p Promotes RCC Progression through HuR-dependent IGF1R/AKT/PI3K Pathway. Cell Death Discov. 7 (1), 147. 10.1038/s41420-021-00525-w 34131104 PMC8206073

[B35] Ham S. Lima L. G. Chai E. P. Z. Muller A. Lobb R. J. Krumeich S. (2018). Breast Cancer-Derived Exosomes Alter Macrophage Polarization via gp130/STAT3 Signaling. Front. Immunol. 9. 10.3389/fimmu.2018.00871 PMC5951966 29867925

[B36] Han X. Huang S. Xue P. Fu J. Liu L. Zhang C. (2019). LncRNAPTPRE-AS1modulates M2 Macrophage Activation and Inflammatory Diseases by Epigenetic Promotion of PTPRE. Sci. Adv. 5 (12), eaax9230. 10.1126/sciadv.aax9230 31844669 PMC6905863

[B37] Hansen T. B. Jensen T. I. Clausen B. H. Bramsen J. B. Finsen B. Damgaard C. K. (2013). Natural RNA Circles Function as Efficient microRNA Sponges. Nature 495 (7441), 384–388. 10.1038/nature11993 23446346

[B38] Hsu D.-H. Paz P. Villaflor G. Rivas A. Mehta-Damani A. Angevin E. (2003). Exosomes as a Tumor Vaccine: Enhancing Potency through Direct Loading of Antigenic Peptides. J. Immunother. 26 (5), 440–450. 10.1097/00002371-200309000-00007 12973033

[B39] Hu Z. Q. Zhou S. L. Li J. Zhou Z. J. Wang P. C. Xin H. Y. (2020). Circular RNA Sequencing Identifies CircASAP1 as a Key Regulator in Hepatocellular Carcinoma Metastasis. Hepatology 72, 906–922. 10.1002/hep.31068 31838741

[B40] Iwai K. Minamisawa T. Suga K. Yajima Y. Shiba K. (2016). Isolation of Human Salivary Extracellular Vesicles by Iodixanol Density Gradient Ultracentrifugation and Their Characterizations. J. Extracellular Vesicles 5, 30829. 10.3402/jev.v5.30829 27193612 PMC4871899

[B41] Jang J.-Y. Lee J.-K. Jeon Y.-K. Kim C.-W. (2013). Exosome Derived from Epigallocatechin Gallate Treated Breast Cancer Cells Suppresses Tumor Growth by Inhibiting Tumor-Associated Macrophage Infiltration and M2 Polarization. BMC Cancer 13, 421. 10.1186/1471-2407-13-421 24044575 PMC3848851

[B42] Jang Y. Kim H. Yoon S. Lee H. Hwang J. Jung J. (2021). Exosome-based Photoacoustic Imaging Guided Photodynamic and Immunotherapy for the Treatment of Pancreatic Cancer. J. Controlled Release 330, 293–304. 10.1016/j.jconrel.2020.12.039 33359580

[B43] Jeppesen D. K. Fenix A. M. Franklin J. L. Higginbotham J. N. Zhang Q. Zimmerman L. J. (2019). Reassessment of Exosome Composition. Cell 177 (2), 428–445. 10.1016/j.cell.2019.02.029 30951670 PMC6664447

[B44] Jurj A. Zanoaga O. Braicu C. Lazar V. Tomuleasa C. Irimie A. (2020). A Comprehensive Picture of Extracellular Vesicles and Their Contents. Molecular Transfer to Cancer Cells. Cancers 12 (2), 298. 10.3390/cancers12020298 PMC7072213 32012717

[B45] Kalluri R. LeBleu V. S. (2020). The Biology, Function, and Biomedical Applications of Exosomes. Science 367 (6478), eaau6977. 10.1126/science.aau6977 32029601 PMC7717626

[B46] Kamerkar S. LeBleu V. S. Sugimoto H. Yang S. Ruivo C. F. Melo S. A. (2017). Exosomes Facilitate Therapeutic Targeting of Oncogenic KRAS in Pancreatic Cancer. Nature 546 (7659), 498–503. 10.1038/nature22341 28607485 PMC5538883

[B47] Kanlikilicer P. Bayraktar R. Denizli M. Rashed M. H. Ivan C. Aslan B. (2018). Exosomal miRNA Confers Chemo Resistance via Targeting Cav1/p-gp/M2-type Macrophage axis in Ovarian Cancer. EBioMedicine 38, 100–112. 10.1016/j.ebiom.2018.11.004 30487062 PMC6306310

[B48] Khalife J. Ghose J. Martella M. Viola D. Rocci A. Troadec E. (2019). MiR-16 Regulates Crosstalk in NF-Κb Tolerogenic Inflammatory Signaling between Myeloma Cells and Bone Marrow Macrophages. JCI Insight 4 (21). 10.1172/jci.insight.129348 PMC6948777 31593552

[B49] Kim M. S. Haney M. J. Zhao Y. Yuan D. Deygen I. Klyachko N. L. (2018). Engineering Macrophage-Derived Exosomes for Targeted Paclitaxel Delivery to Pulmonary Metastases: *In Vitro* and *In Vivo* Evaluations. Nanomedicine: Nanotechnology, Biol. Med. 14 (1), 195–204. 10.1016/j.nano.2017.09.011 28982587

[B50] Klinge C. (2018). Non-Coding RNAs in Breast Cancer: Intracellular and Intercellular Communication. ncRNA 4 (4), 40. 10.3390/ncrna4040040 PMC6316884 30545127

[B51] Kumar S. R. Kimchi E. T. Manjunath Y. Gajagowni S. Stuckel A. J. Kaifi J. T. (2020). RNA Cargos in Extracellular Vesicles Derived from Blood Serum in Pancreas Associated Conditions. Sci. Rep. 10 (1), 2800. 10.1038/s41598-020-59523-0 32071328 PMC7028741

[B52] Lan B. Zeng S. Grützmann R. Pilarsky C. (2019a). The Role of Exosomes in Pancreatic Cancer. Ijms 20 (18), 4332. 10.3390/ijms20184332 PMC6770781 31487880

[B53] Lan J. Sun L. Xu F. Liu L. Hu F. Song D. (2019b). M2 Macrophage-Derived Exosomes Promote Cell Migration and Invasion in Colon Cancer. Cancer Res. 79 (1), 146–158. 10.1158/0008-5472.CAN-18-0014 30401711

[B54] Lawrence T. Natoli G. (2011). Transcriptional Regulation of Macrophage Polarization: Enabling Diversity with Identity. Nat. Rev. Immunol. 11 (11), 750–761. 10.1038/nri3088 22025054

[B55] Lee H. D. Koo B. H. Kim Y. H. Jeon O. H. Kim D. S. (2012). Exosome Release of ADAM15 and the Functional Implications of Human Macrophage‐derived ADAM15 Exosomes. FASEB j. 26 (7), 3084–3095. 10.1096/fj.11-201681 22505472

[B56] Leone D. A. Rees A. J. Kain R. (2018). Dendritic Cells and Routing Cargo into Exosomes. Immunol. Cel Biol 96, 683–693. 10.1111/imcb.12170 29797348

[B57] Li B. Song T.-N. Wang F.-R. Yin C. Li Z. Lin J.-P. (2019). Tumor-derived Exosomal HMGB1 Promotes Esophageal Squamous Cell Carcinoma Progression through Inducing PD1+ TAM Expansion. Oncogenesis 8 (3), 17. 10.1038/s41389-019-0126-2 30796203 PMC6386749

[B58] Li H. Yang C. Shi Y. Zhao L. (2018a). Exosomes Derived from siRNA against GRP78 Modified Bone-Marrow-Derived Mesenchymal Stem Cells Suppress Sorafenib Resistance in Hepatocellular Carcinoma. J. Nanobiotechnol 16 (1), 103. 10.1186/s12951-018-0429-z PMC6300915 30572882

[B59] Li T. Sun X. Chen L. (2020a). Exosome Circ_0044516 Promotes Prostate Cancer Cell Proliferation and Metastasis as a Potential Biomarker. J. Cel Biochem 121 (3), 2118–2126. 10.1002/jcb.28239 31625175

[B60] Li X. Lei Y. Wu M. Li N. (2018b). Regulation of Macrophage Activation and Polarization by HCC-Derived Exosomal lncRNA TUC339. Ijms 19 (10), 2958. 10.3390/ijms19102958 PMC6213212 30274167

[B61] Li Z. Suo B. Long G. Gao Y. Song J. Zhang M. (2020b). Exosomal miRNA-16-5p Derived from M1 Macrophages Enhances T Cell-dependent Immune Response by Regulating PD-L1 in Gastric Cancer. Front. Cel Dev. Biol. 8, 572689. 10.3389/fcell.2020.572689 PMC7734296 33330451

[B62] Liang Y. Song X. Li Y. Chen B. Zhao W. Wang L. (2020). LncRNA BCRT1 Promotes Breast Cancer Progression by Targeting miR-1303/PTBP3 axis. Mol. Cancer 19 (1), 85. 10.1186/s12943-020-01206-5 32384893 PMC7206728

[B63] Liang Z.-x. Liu H.-s. Wang F.-w. Xiong L. Zhou C. Hu T. (2019). LncRNA RPPH1 Promotes Colorectal Cancer Metastasis by Interacting with TUBB3 and by Promoting Exosomes-Mediated Macrophage M2 Polarization. Cell Death Dis 10 (11), 829. 10.1038/s41419-019-2077-0 31685807 PMC6828701

[B64] Lin Y. Xu J. Lan H. (2019). Tumor-associated Macrophages in Tumor Metastasis: Biological Roles and Clinical Therapeutic Applications. J. Hematol. Oncol. 12 (1), 76. 10.1186/s13045-019-0760-3 31300030 PMC6626377

[B65] Liu G. Luo Y. Hou P. (2021a). PRPS2 Enhances Resistance to Cisplatin via Facilitating Exosomes-Mediated Macrophage M2 Polarization in Non-small Cell Lung Cancer. Immunological Invest., 1–14. 10.1080/08820139.2021.1952217 34251965

[B66] Liu J. Fan L. Yu H. Zhang J. He Y. Feng D. (2019). Endoplasmic Reticulum Stress Causes Liver Cancer Cells to Release Exosomal miR‐23a‐3p and Up‐regulate Programmed Death Ligand 1 Expression in Macrophages. Hepatology 70 (1), 241–258. 10.1002/hep.30607 30854665 PMC6597282

[B67] Liu T. Zhang X. Gao S. Jing F. Yang Y. Du L. (2016). Exosomal Long Noncoding RNA CRNDE-H as a Novel Serum-Based Biomarker for Diagnosis and Prognosis of Colorectal Cancer. Oncotarget 7 (51), 85551–85563. 10.18632/oncotarget.13465 27888803 PMC5356757

[B68] Liu W. Long Q. Zhang W. Zeng D. Hu B. Liu S. (2021b). miRNA-221-3p Derived from M2-Polarized Tumor-Associated Macrophage Exosomes Aggravates the Growth and Metastasis of Osteosarcoma through SOCS3/JAK2/STAT3 axis. Aging 13 (undefined), 19760–19775. 10.18632/aging.203388 34388111 PMC8386545

[B69] Lu J. Wang Y.-h. Yoon C. Huang X.-y. Xu Y. Xie J.-w. (2020). Circular RNA Circ-RanGAP1 Regulates VEGFA Expression by Targeting miR-877-3p to Facilitate Gastric Cancer Invasion and Metastasis. Cancer Lett. 471, 38–48. 10.1016/j.canlet.2019.11.038 31811909

[B70] Ma Y.-S. Wu T.-M. Ling C.-C. Yu F. Zhang J. Cao P.-S. (2021). M2 Macrophage-Derived Exosomal microRNA-155-5p Promotes the Immune Escape of colon Cancer by Downregulating ZC3H12B. Mol. Ther. - Oncolytics 20, 484–498. 10.1016/j.omto.2021.02.005 33718596 PMC7932913

[B71] Maia J. Caja S. Strano Moraes M. C. Couto N. Costa-Silva B. (2018). Exosome-Based Cell-Cell Communication in the Tumor Microenvironment. Front. Cel Dev. Biol. 6, 18. 10.3389/fcell.2018.00018 PMC5826063 29515996

[B72] Makarova J. Turchinovich A. Shkurnikov M. Tonevitsky A. (2021). Extracellular miRNAs and Cell-Cell Communication: Problems and Prospects. Trends Biochem. Sci. 46 (8), 640–651. 10.1016/j.tibs.2021.01.007 33610425

[B73] Mantovani A. Marchesi F. Malesci A. Laghi L. Allavena P. (2017). Tumour-associated Macrophages as Treatment Targets in Oncology. Nat. Rev. Clin. Oncol. 14 (7), 399–416. 10.1038/nrclinonc.2016.217 28117416 PMC5480600

[B74] Mantovani A. Sozzani S. Locati M. Allavena P. Sica A. (2002). Macrophage Polarization: Tumor-Associated Macrophages as a Paradigm for Polarized M2 Mononuclear Phagocytes. Trends Immunol. 23 (11), 549–555. 10.1016/s1471-4906(02)02302-5 12401408

[B75] Markov O. Oshchepkova A. Mironova N. (2019). Immunotherapy Based on Dendritic Cell-Targeted/-Derived Extracellular Vesicles-A Novel Strategy for Enhancement of the Anti-tumor Immune Response. Front. Pharmacol. 10, 1152. 10.3389/fphar.2019.01152 31680949 PMC6798004

[B76] McKenzie A. J. Hoshino D. Hong N. H. Cha D. J. Franklin J. L. Coffey R. J. (2016). KRAS-MEK Signaling Controls Ago2 Sorting into Exosomes. Cel Rep. 15 (5), 978–987. 10.1016/j.celrep.2016.03.085 PMC4857875 27117408

[B77] Meldolesi J. (2018). Exosomes and Ectosomes in Intercellular Communication. Curr. Biol. 28 (8), R435–R444. 10.1016/j.cub.2018.01.059 29689228

[B78] Mi X. Xu R. Hong S. Xu T. Zhang W. Liu M. (2020). M2 Macrophage-Derived Exosomal lncRNA AFAP1-AS1 and MicroRNA-26a Affect Cell Migration and Metastasis in Esophageal Cancer. Mol. Ther. - Nucleic Acids 22, 779–790. 10.1016/j.omtn.2020.09.035 33230475 PMC7595846

[B79] Moradi-Chaleshtori M. Bandehpour M. Heidari N. Mohammadi-Yeganeh S. Mahmoud Hashemi S. (2021a). Exosome-mediated miR-33 Transfer Induces M1 Polarization in Mouse Macrophages and Exerts Antitumor Effect in 4T1 Breast Cancer Cell Line. Int. Immunopharmacology 90, 107198. 10.1016/j.intimp.2020.107198 33249048

[B80] Moradi-Chaleshtori M. Bandehpour M. Soudi S. Mohammadi-Yeganeh S. Hashemi S. M. (2021b). *In Vitro* and *In Vivo* Evaluation of Anti-tumoral Effect of M1 Phenotype Induction in Macrophages by miR-130 and miR-33 Containing Exosomes. Cancer Immunol. Immunother. 70 (5), 1323–1339. 10.1007/s00262-020-02762-x 33140190 PMC10991174

[B81] Nabet B. Y. Qiu Y. Shabason J. E. Wu T. J. Yoon T. Kim B. C. (2017). Exosome RNA Unshielding Couples Stromal Activation to Pattern Recognition Receptor Signaling in Cancer. Cell 170, 352–366. 10.1016/j.cell.2017.06.031 28709002 PMC6611169

[B82] Naseri M. Bozorgmehr M. Zöller M. Ranaei Pirmardan E. Madjd Z. (2020). Tumor-derived Exosomes: the Next Generation of Promising Cell-free Vaccines in Cancer Immunotherapy. Oncoimmunology 9 (1), 1779991. 10.1080/2162402x.2020.1779991 32934883 PMC7466856

[B83] O’Brien K. P. Khan S. Gilligan K. E. Zafar H. Lalor P. Glynn C. (2018). Employing Mesenchymal Stem Cells to Support Tumor-Targeted Delivery of Extracellular Vesicle (EV)-encapsulated microRNA-379. Oncogene 37 (16), 2137–2149. 10.1038/s41388-017-0116-9 29367765

[B84] Orecchioni M. Ghosheh Y. Pramod A. B. Ley K. (2019). Macrophage Polarization: Different Gene Signatures in M1(LPS+) vs. Classically and M2(LPS-) vs. Alternatively Activated Macrophages. Front. Immunol. 10, 1084. 10.3389/fimmu.2019.01084 31178859 PMC6543837

[B85] Pang X. Wang S.-s. Zhang M. Jiang J. Fan H.-y. Wu J.-s. (2021). OSCC Cell-Secreted Exosomal CMTM6 Induced M2-like Macrophages Polarization via ERK1/2 Signaling Pathway. Cancer Immunol. Immunother. 70 (4), 1015–1029. 10.1007/s00262-020-02741-2 33104837 PMC10991130

[B86] Petty A. J. Yang Y. (2017). Tumor-associated Macrophages: Implications in Cancer Immunotherapy. Immunotherapy 9 (3), 289–302. 10.2217/imt-2016-0135 28231720 PMC5619052

[B87] Popēna I. Ābols A. Saulīte L. Pleiko K. Zandberga E. Jēkabsons K. (2018). Effect of Colorectal Cancer-Derived Extracellular Vesicles on the Immunophenotype and Cytokine Secretion Profile of Monocytes and Macrophages. Cell Commun Signal 16 (1), 17. 10.1186/s12964-018-0229-y 29690889 PMC5937830

[B88] Purushothaman A. Bandari S. K. Chandrashekar D. S. Jones R. J. Lee H. C. Weber D. M. (2017). Chondroitin Sulfate Proteoglycan Serglycin Influences Protein Cargo Loading and Functions of Tumor-Derived Exosomes. Oncotarget 8 (43), 73723–73732. 10.18632/oncotarget.20564 29088739 PMC5650294

[B89] Qian M. Wang S. Guo X. Wang J. Zhang Z. Qiu W. (2020). Hypoxic Glioma-Derived Exosomes Deliver microRNA-1246 to Induce M2 Macrophage Polarization by Targeting TERF2IP via the STAT3 and NF-Κb Pathways. Oncogene 39 (2), 428–442. 10.1038/s41388-019-0996-y 31485019

[B90] Record M. Silvente-Poirot S. Poirot M. Wakelam M. O. (2018). Extracellular Vesicles: Lipids as Key Components of Their Biogenesis and Functions. J. Lipid Res. 59 (8), 1316–1324. 10.1194/jlr.E086173 29764923 PMC6071772

[B91] Roy S. Bag A. K. Dutta S. Polavaram N. S. Islam R. Schellenburg S. (2018). Macrophage-Derived Neuropilin-2 Exhibits Novel Tumor-Promoting Functions. Cancer Res. 78 (19), 5600–5617. 10.1158/0008-5472.can-18-0562 30111533 PMC6168405

[B92] Ruffell B. Affara N. I. Coussens L. M. (2012). Differential Macrophage Programming in the Tumor Microenvironment. Trends Immunology 33 (3), 119–126. 10.1016/j.it.2011.12.001 PMC3294003 22277903

[B93] Schwarzenbach H. Gahan P. B. (2021). Exosomes in Immune Regulation. ncRNA 7 (1), 4. 10.3390/ncrna7010004 33435564 PMC7838779

[B94] Seo N. Shirakura Y. Tahara Y. Momose F. Harada N. Ikeda H. (2018). Activated CD8+ T Cell Extracellular Vesicles Prevent Tumour Progression by Targeting of Lesional Mesenchymal Cells. Nat. Commun. 9 (1), 435. 10.1038/s41467-018-02865-1 29382847 PMC5789986

[B95] Shapouri‐Moghaddam A. Mohammadian S. Vazini H. Taghadosi M. Esmaeili S. A. Mardani F. (2018). Macrophage Plasticity, Polarization, and Function in Health and Disease. J. Cel Physiol 233 (9), 6425–6440. 10.1002/jcp.26429 PMC13482560 29319160

[B96] Shinohara H. Kuranaga Y. Kumazaki M. Sugito N. Yoshikawa Y. Takai T. (2017). Regulated Polarization of Tumor-Associated Macrophages by miR-145 via Colorectal Cancer-Derived Extracellular Vesicles. J.I. 199 (4), 1505–1515. 10.4049/jimmunol.1700167 28696255

[B97] Soki F. N. Koh A. J. Jones J. D. Kim Y. W. Dai J. Keller E. T. (2014). Polarization of Prostate Cancer-Associated Macrophages Is Induced by Milk Fat Globule-EGF Factor 8 (MFG-E8)-Mediated Efferocytosis. J. Biol. Chem. 289 (35), 24560–24572. 10.1074/jbc.M114.571620 25006249 PMC4148880

[B98] Steinbichler T. B. Dudás J. Skvortsov S. Ganswindt U. Riechelmann H. Skvortsova II (2019). Therapy Resistance Mediated by Exosomes. Mol. Cancer 18 (1), 58. 10.1186/s12943-019-0970-x 30925921 PMC6441190

[B99] Su M.-J. Aldawsari H. Amiji M. (2016). Pancreatic Cancer Cell Exosome-Mediated Macrophage Reprogramming and the Role of MicroRNAs 155 and 125b2 Transfection Using Nanoparticle Delivery Systems. Sci. Rep. 6, 30110. 10.1038/srep30110 27443190 PMC4957091

[B100] Sung B. H. Ketova T. Hoshino D. Zijlstra A. Weaver A. M. (2015). Directional Cell Movement through Tissues Is Controlled by Exosome Secretion. Nat. Commun. 6, 7164. 10.1038/ncomms8164 25968605 PMC4435734

[B101] Takano Y. Masuda T. Iinuma H. Yamaguchi R. Sato K. Tobo T. (2017). Circulating Exosomal microRNA-203 Is Associated with Metastasis Possibly via Inducing Tumor-Associated Macrophages in Colorectal Cancer. Oncotarget 8 (45), 78598–78613. 10.18632/oncotarget.20009 29108252 PMC5667985

[B102] Tang Y.-T. Huang Y.-Y. Zheng L. Qin S.-H. Xu X.-P. An T.-X. (2017). Comparison of Isolation Methods of Exosomes and Exosomal RNA from Cell Culture Medium and Serum. Int. J. Mol. Med. 40 (3), 834–844. 10.3892/ijmm.2017.3080 28737826 PMC5548045

[B103] Turchinovich A. Weiz L. Langheinz A. Burwinkel B. (2011). Characterization of Extracellular Circulating microRNA. Nucleic Acids Res. 39 (16), 7223–7233. 10.1093/nar/gkr254 21609964 PMC3167594

[B104] Vlassov A. V. Magdaleno S. Setterquist R. Conrad R. (2012). Exosomes: Current Knowledge of Their Composition, Biological Functions, and Diagnostic and Therapeutic Potentials. Biochim. Biophys. Acta (Bba) - Gen. Subjects 1820 (7), 940–948. 10.1016/j.bbagen.2012.03.017 22503788

[B105] Wang K. Zhang S. Weber J. Baxter D. Galas D. J. (2010). Export of microRNAs and microRNA-Protective Protein by Mammalian Cells. Nucleic Acids Res. 38 (20), 7248–7259. 10.1093/nar/gkq601 20615901 PMC2978372

[B106] Wang X. Luo G. Zhang K. Cao J. Huang C. Jiang T. (2018). Hypoxic Tumor-Derived Exosomal miR-301a Mediates M2 Macrophage Polarization via PTEN/PI3Kγ to Promote Pancreatic Cancer Metastasis. Cancer Res. 78 (16), 4586–4598. 10.1158/0008-5472.can-17-3841 29880482

[B107] Wang Y. Gao R. Li J. Tang S. Li S. Tong Q. (2021). Downregulation of Hsa_circ_0074854 Suppresses the Migration and Invasion in Hepatocellular Carcinoma via Interacting with HuR and via Suppressing Exosomes-Mediated Macrophage M2 Polarization. Ijn 16, 2803–2818. 10.2147/ijn.s284560 33880025 PMC8052130

[B108] Wu J. Gao W. Tang Q. Yu Y. You W. Wu Z. (2021). M2 Macrophage-Derived Exosomes Facilitate HCC Metastasis by Transferring α M β 2 Integrin to Tumor Cells. Hepatology 73 (4), 1365–1380. 10.1002/hep.31432 32594528 PMC8360085

[B109] Wu K. Lin K. Li X. Yuan X. Xu P. Ni P. (2020). Redefining Tumor-Associated Macrophage Subpopulations and Functions in the Tumor Microenvironment. Front. Immunol. 11, 1731. 10.3389/fimmu.2020.01731 32849616 PMC7417513

[B110] Xiao L. Erb U. Zhao K. Hackert T. Zöller M. (2017). Efficacy of Vaccination with Tumor-Exosome Loaded Dendritic Cells Combined with Cytotoxic Drug Treatment in Pancreatic Cancer. Oncoimmunology 6 (6), e1319044. 10.1080/2162402x.2017.1319044 28680753 PMC5486185

[B111] Xie Y. Wu J. Xu A. Ahmeqd S. Sami A. Chibbar R. (2018). Heterologous Human/rat HER2-specific Exosome-Targeted T Cell Vaccine Stimulates Potent Humoral and CTL Responses Leading to Enhanced Circumvention of HER2 Tolerance in Double Transgenic HLA-A2/her2 Mice. Vaccine 36 (11), 1414–1422. 10.1016/j.vaccine.2018.01.078 29415817

[B112] Xu R. Greening D. W. Rai A. Ji H. Simpson R. J. (2015). Highly-purified Exosomes and Shed Microvesicles Isolated from the Human colon Cancer Cell Line LIM1863 by Sequential Centrifugal Ultrafiltration Are Biochemically and Functionally Distinct. Methods 87, 11–25. 10.1016/j.ymeth.2015.04.008 25890246

[B113] Xu Y. Wu W. Han Q. Wang Y. Li C. Zhang P. (2019). New Insights into the Interplay between Non-coding RNAs and RNA-Binding Protein HnRNPK in Regulating Cellular Functions. Cells 8 (1), 62. 10.3390/cells8010062 PMC6357021 30658384

[B114] Yang B. Teng F. Chang L. Wang J. Liu D.-L. Cui Y.-S. (2021a). Tumor-derived Exosomal circRNA_102481 Contributes to EGFR-TKIs Resistance via the miR-30a-5p/ROR1 axis in Non-small Cell Lung Cancer. Aging 13 (9), 13264–13286. 10.18632/aging.203011 33952725 PMC8148492

[B115] Yang L. Dong Y. Li Y. Wang D. Liu S. Wang D. (2019a). IL‐10 Derived from M2 Macrophage Promotes Cancer Stemness via JAK1/STAT1/NF‐κB/Notch1 Pathway in Non‐small Cell Lung Cancer. Int. J. Cancer 145 (4), 1099–1110. 10.1002/ijc.32151 30671927

[B116] Yang M. Chen J. Su F. Yu B. Su F. Lin L. (2011). Microvesicles Secreted by Macrophages Shuttle Invasion-Potentiating microRNAs into Breast Cancer Cells. Mol. Cancer 10, 117. 10.1186/1476-4598-10-117 21939504 PMC3190352

[B117] Yang X.-X. Sun C. Wang L. Guo X.-L. (2019b). New Insight into Isolation, Identification Techniques and Medical Applications of Exosomes. J. Controlled Release 308, 119–129. 10.1016/j.jconrel.2019.07.021 31325471

[B118] Yang Y. Guo Z. Chen W. Wang X. Cao M. Han X. (2021b). M2 Macrophage-Derived Exosomes Promote Angiogenesis and Growth of Pancreatic Ductal Adenocarcinoma by Targeting E2F2. Mol. Ther. 29 (3), 1226–1238. 10.1016/j.ymthe.2020.11.024 33221435 PMC7934635

[B119] Yin C. Han Q. Xu D. Zheng B. Zhao X. Zhang J. (2019a). SALL4-mediated Upregulation of Exosomal miR-146a-5p Drives T-Cell Exhaustion by M2 Tumor-Associated Macrophages in HCC. Oncoimmunology 8 (7), e1601479. 10.1080/2162402x.2019.1601479 PMC6527304 31143524

[B120] Yin Z. Ma T. Huang B. Lin L. Zhou Y. Yan J. (2019b). Macrophage-derived Exosomal microRNA-501-3p Promotes Progression of Pancreatic Ductal Adenocarcinoma through the TGFBR3-Mediated TGF-β Signaling Pathway. J. Exp. Clin. Cancer Res. 38 (1), 310. 10.1186/s13046-019-1313-x 31307515 PMC6631643

[B121] Yin Z. Zhou Y. Ma T. Chen S. Shi N. Zou Y. (2020). Down‐regulated lncRNA SBF2‐AS1 in M2 Macrophage‐derived Exosomes Elevates miR‐122‐5p to Restrict XIAP, Thereby Limiting Pancreatic Cancer Development. J. Cel Mol Med 24 (9), 5028–5038. 10.1111/jcmm.15125 PMC7205800 32301277

[B122] Ying X. Wu Q. Wu X. Zhu Q. Wang X. Jiang L. (2016). Epithelial Ovarian Cancer-Secreted Exosomal miR-222-3p Induces Polarization of Tumor-Associated Macrophages. Oncotarget 7 (28), 43076–43087. 10.18632/oncotarget.9246 27172798 PMC5190009

[B123] Zhan Q. Yi K. Qi H. Li S. Li X. Wang Q. (2020). Engineering Blood Exosomes for Tumor-Targeting Efficient Gene/chemo Combination Therapy. Theranostics 10 (17), 7889–7905. 10.7150/thno.45028 32685027 PMC7359100

[B124] Zhang F. Sang Y. Chen D. Wu X. Wang X. Yang W. (2021a). M2 Macrophage-Derived Exosomal Long Non-coding RNA AGAP2-AS1 Enhances Radiotherapy Immunity in Lung Cancer by Reducing microRNA-296 and Elevating NOTCH2. Cel Death Dis 12 (5), 467. 10.1038/s41419-021-03700-0 PMC8110970 33972506

[B125] Zhang H. Yu Y. Wang J. Han Y. Ren T. Huang Y. (2021b). Macrophages-derived Exosomal lncRNA LIFR-AS1 Promotes Osteosarcoma Cell Progression via miR-29a/NFIA axis. Cancer Cel Int 21 (1), 192. 10.1186/s12935-021-01893-0 PMC8017664 33794884

[B126] Zhang W.-J. Wang X.-H. Gao S.-T. Chen C. Xu X.-Y. Sun Q. (2018). Tumor-associated Macrophages Correlate with Phenomenon of Epithelial-Mesenchymal Transition and Contribute to Poor Prognosis in Triple-Negative Breast Cancer Patients. J. Surg. Res. 222, 93–101. 10.1016/j.jss.2017.09.035 29273380

[B127] Zhang X. Sai B. Wang F. Wang L. Wang Y. Zheng L. (2019). Hypoxic BMSC-Derived Exosomal miRNAs Promote Metastasis of Lung Cancer Cells via STAT3-Induced EMT. Mol. Cancer 18 (1), 40. 10.1186/s12943-019-0959-5 30866952 PMC6417285

[B128] Zhang Y. Bi J. Huang J. Tang Y. Du S. Li P. (2020). Exosome: A Review of its Classification, Isolation Techniques, Storage, Diagnostic and Targeted Therapy Applications. Ijn 15, 6917–6934. 10.2147/ijn.s264498 33061359 PMC7519827

[B129] Zhang Y. Zhang Y. Li X. Zhang M. Lv K. (2017). Microarray Analysis of Circular RNA Expression Patterns in Polarized Macrophages. Int. J. Mol. Med. 39 (2), 373–379. 10.3892/ijmm.2017.2852 28075448 PMC5358696

[B130] Zhang Z. Lu M. Qin Y. Gao W. Tao L. Su W. (2021c). Neoantigen: A New Breakthrough in Tumor Immunotherapy. Front. Immunol. 12, 672356. 10.3389/fimmu.2021.672356 33936118 PMC8085349

[B131] Zhao S. Mi Y. Guan B. Zheng B. Wei P. Gu Y. (2020). Tumor-derived Exosomal miR-934 Induces Macrophage M2 Polarization to Promote Liver Metastasis of Colorectal Cancer. J. Hematol. Oncol. 13 (1), 156. 10.1186/s13045-020-00991-2 33213490 PMC7678301

[B132] Zhao Y. Ge X. Xu X. Yu S. Wang J. Sun L. (2019a). Prognostic Value and Clinicopathological Roles of Phenotypes of Tumour-Associated Macrophages in Colorectal Cancer. J. Cancer Res. Clin. Oncol. 145 (12), 3005–3019. 10.1007/s00432-019-03041-8 31650222 PMC11810290

[B133] Zhao Z. Ji M. Wang Q. He N. Li Y. (2019b). Circular RNA Cdr1as Upregulates SCAI to Suppress Cisplatin Resistance in Ovarian Cancer via miR-1270 Suppression. Mol. Ther. - Nucleic Acids 18, 24–33. 10.1016/j.omtn.2019.07.012 31479922 PMC6726918

[B134] Zhao Z. Wijerathne H. Godwin A. K. Soper S. A. (2021). Isolation and Analysis Methods of Extracellular Vesicles (EVs). Evcna 2, 80–103. 10.20517/evcna.2021.07 34414401 PMC8372011

[B135] Zheng P. Chen L. Yuan X. Luo Q. Liu Y. Xie G. (2017). Exosomal Transfer of Tumor-Associated Macrophage-Derived miR-21 Confers Cisplatin Resistance in Gastric Cancer Cells. J. Exp. Clin. Cancer Res. 36 (1), 53. 10.1186/s13046-017-0528-y 28407783 PMC5390430

[B136] Zheng P. Luo Q. Wang W. Li J. Wang T. Wang P. (2018). Tumor-associated Macrophages-Derived Exosomes Promote the Migration of Gastric Cancer Cells by Transfer of Functional Apolipoprotein E. Cel Death Dis 9 (4), 434. 10.1038/s41419-018-0465-5 PMC5864742 29567987

[B137] Zhong L. Liao D. Li J. Liu W. Wang J. Zeng C. (2021). Rab22a-NeoF1 Fusion Protein Promotes Osteosarcoma Lung Metastasis through its Secretion into Exosomes. Sig Transduct Target. Ther. 6 (1), 59. 10.1038/s41392-020-00414-1 PMC7876000 33568623

[B138] Zhou C.-F. Ma J. Huang L. Yi H.-Y. Zhang Y.-M. Wu X.-G. (2019). Cervical Squamous Cell Carcinoma-Secreted Exosomal miR-221-3p Promotes Lymphangiogenesis and Lymphatic Metastasis by Targeting VASH1. Oncogene 38 (8), 1256–1268. 10.1038/s41388-018-0511-x 30254211 PMC6363643

[B139] Zhou D. Xia Z. Xie M. Gao Y. Yu Q. He B. (2021). Exosomal Long Non-coding RNA SOX2 Overlapping Transcript Enhances the Resistance to EGFR-TKIs in Non-small Cell Lung Cancer Cell Line H1975. Hum. Cel 34 (5), 1478–1489. 10.1007/s13577-021-00572-6 34244990

[B140] Zhou J.-h. Yao Z.-x. Zheng Z. Yang J. Wang R. Fu S.-j. (2020). G-MDSCs-Derived Exosomal miRNA-143-3p Promotes Proliferation via Targeting of ITM2B in Lung Cancer. Ott 13, 9701–9719. 10.2147/ott.s256378 PMC7533249 33061450

[B141] Zhou Y. Ren H. Dai B. Li J. Shang L. Huang J. (2018). Hepatocellular Carcinoma-Derived Exosomal miRNA-21 Contributes to Tumor Progression by Converting Hepatocyte Stellate Cells to Cancer-Associated Fibroblasts. J. Exp. Clin. Cancer Res. 37 (1), 324. 10.1186/s13046-018-0965-2 30591064 PMC6307162

[B142] Zhu L. Sun H.-T. Wang S. Huang S.-L. Zheng Y. Wang C.-Q. (2020). Isolation and Characterization of Exosomes for Cancer Research. J. Hematol. Oncol. 13 (1), 152. 10.1186/s13045-020-00987-y 33168028 PMC7652679

[B143] Zhu X. Shen H. Yin X. Yang M. Wei H. Chen Q. (2019). Macrophages Derived Exosomes Deliver miR-223 to Epithelial Ovarian Cancer Cells to Elicit a Chemoresistant Phenotype. J. Exp. Clin. Cancer Res. 38 (1), 81. 10.1186/s13046-019-1095-1 30770776 PMC6377760

[B144] Zhu Y. Chen X. Pan Q. Wang Y. Su S. Jiang C. (2015). A Comprehensive Proteomics Analysis Reveals a Secretory Path- and Status-dependent Signature of Exosomes Released from Tumor-Associated Macrophages. J. Proteome Res. 14 (10), 4319–4331. 10.1021/acs.jproteome.5b00770 26312558

